# Analysis of Social Mission Commitment at Dental, Medical, and Nursing Schools in the US

**DOI:** 10.1001/jamanetworkopen.2022.10900

**Published:** 2022-05-09

**Authors:** Sonal Batra, Julie Orban, Hexuan Zhang, Thomas M. Guterbock, Leigh Anne Butler, Colleen Bogucki, Candice Chen

**Affiliations:** 1 School of Medicine and Health Sciences, The George Washington University, Washington, District of Columbia; 2 Milken Institute School of Public Health, The George Washington University, Washington, District of Columbia; 3 Charles River Associates, Summit, New Jersey; 4 Center for Survey Research, University of Virginia, Charlottesville

## Abstract

**Importance:**

The COVID-19 pandemic and calls for racial justice have highlighted the need for schools to promote social mission. Measuring social mission engagement and performance in health professions education may encourage institutional efforts to advance health equity and social justice commitments.

**Objective:**

To describe the current state of social mission commitment within dental, medical, and nursing schools in the US and to examine how social mission performance compares across school types.

**Design, Setting, and Participants:**

This cross-sectional survey study invited all US dental and medical schools and a subset of baccalaureate- and master’s degree–conferring nursing schools to participate in a self-assessment to measure their school’s social mission commitment from January 29 through October 9, 2019. The survey measured 79 indicators (with indicators defined as responses to specific scored questions that indicated the state or level of social mission commitment) across 18 areas in 6 domains of school functioning (educational program, community engagement, governance, diversity and inclusion, institutional culture and climate, and research) that have potential to enhance social mission engagement and performance. Individual health professions schools were the unit of analysis, and 689 dental, medical, and nursing schools were invited to participate. School deans and program directors were the primary target respondents because of their broad insight into their school’s programs and policies and their ability to request data from various internal sources. Demographic information from respondents was not collected because multiple respondents from an institution could complete different sections of the survey.

**Main Outcomes and Measures:**

Survey responses were analyzed to create indicator scores, standardized area scores, and an overall social mission score for each school. Using descriptive analyses, frequency and contingency tables of specific indicators within each area were created, and schools were compared based on ownership status (private or public), Carnegie Classification of Institutions of Higher Education research classification group (doctoral university with very high research activity [R1], doctoral university with high [R2] or moderate [R3] research activity, baccalaureate or master’s nursing college or university, or special focus institution), and discipline group (dental school, medical school granting doctor of osteopathic medicine [DO] degrees, medical school granting doctor of medicine [MD] degrees, nursing school granting baccalaureate-level degrees, or nursing school granting master’s-level degrees).

**Results:**

Among 689 invited schools, 242 schools (35.1%) completed the self-assessment survey. Of those, 133 (55.0%) were nursing schools, 83 (34.3%) were medical schools, and 26 (10.7%) were dental schools. Response rates ranged from 133 of 420 invited nursing schools (31.7%) to 83 of 203 invited medical schools (40.9%). Most schools included social determinants of health in their curriculum in either required courses (233 of 242 schools [96.3%]) or elective courses (4 of 242 schools [1.7%]), but only 116 of 235 schools (49.4%) integrated social determinants of health across all years of study. Most schools also included health disparities in either their required courses (232 of 242 [95.9%]) or elective courses (6 of 242 [2.5%]); however, only 118 of 235 schools (50.2%) integrated health disparities across all years of study. In several areas of social mission, public schools performed better than private schools (eg, curriculum: mean [SE] standardized area score, 0.13 [0.07] points vs −0.14 [0.09] points, respectively), and R1 doctoral universities and special focus institutions performed better than R2 and R3 doctoral universities and baccalaureate and master’s nursing colleges and universities (eg, extracurricular activities: mean [SE] standardized area score, 0.25 [0.09] points for R1 doctoral universities and 0.20 [0.12] points for special focus institutions vs −0.05 [0.12] points for R2 and R3 doctoral universities and – 0.30 [0.12] points for baccalaureate and master’s nursing colleges and universities. Different areas of strength emerged for dental, medical, and nursing schools. For example, in the curriculum area, MD-granting medical schools had a mean (SE) standardized area score of 0.38 (0.08) points, which was significantly higher than the standardized area scores of dental schools (mean [SE], −0.21 [0.14] points), DO-granting medical schools (mean [SE], −0.22 [0.13] points), graduate nursing schools (mean [SE], −0.21 [0.19] points), and undergraduate nursing schools (mean [SE], −0.05 [0.10] points).

**Conclusions and Relevance:**

In this study, there was widespread interest from health professions educational leaders in understanding and enhancing social mission commitment. Future work may focus on identifying and promoting best practices using the framework described, providing schools with continued opportunities for self-assessment, and further validating the self-assessment survey.

## Key Points

**Question:**

What is the current state of social mission commitment in US dental, medical, and nursing schools, and how does social mission performance compare across school types?

**Findings:**

In this survey study of 689 dental, medical, and nursing schools, public schools performed better than private schools, and doctoral universities classified as having very high research activity (R1) based on the Carnegie Classification of Institutions of Higher Education and special focus institutions performed better than doctoral universities classified as having high (R2) or moderate (R3) research activity and baccalaureate- and master’s degree–granting nursing colleges and universities in several areas of social mission. Different areas of social mission strength emerged for each discipline.

**Meaning:**

This study’s findings suggest that understanding social mission commitment may aid in developing targeted strategies for enhancing social mission engagement and performance within health professions schools.

## Teaser

This survey study describes the current state of social mission commitment within dental, medical, and nursing schools in the US and examines how social mission engagement and performance compares across school types.

## Introduction

Social mission is the contribution of a school to enhancing health equity and addressing the health disparities of the society in which it exists through its mission, programs, and the performance of its graduates, faculty, and leadership.^1^ The COVID-19 pandemic and calls for racial justice have highlighted the imperative for health professions schools to promote social mission. As disparities are identified and health care continues to evolve, health care professionals will need to be taught to go beyond the basic sciences and individual patient encounters to engage communities, understand the root causes of health inequities, and address the social and structural determinants of health.^2,3,4^ Social mission is more than simply the inclusion of certain topics in a school’s curriculum; it encompasses the mission of the school, the school’s relationship with the community, and structures and policies that have the potential to advance health equity and social justice commitments.^1^

The development of the Social Mission Metrics (SMM) self-assessment for health professions schools has been previously described.^5^ Self-assessment tools are important to helping schools identify strengths and weaknesses and allowing schools to capitalize on the work they are already successfully performing while refining their work in areas needing improvement.

In addition to the value of individual school self-assessment, social mission will need to be understood on a national level to catalyze widespread improvement. Assessing social mission through an established framework allows for the identification of national norms and can highlight schools and programs that serve as exemplars. Assessment also allows for exploring the association between social mission in various activity areas and health equity–related workforce outcomes.^5^ The objective of this survey study was to describe the current state of social mission commitment within dental, medical, and nursing schools in the US and to examine how social mission performance compared across school types.

## Methods

### School Recruitment

In this cross-sectional survey study, all US dental, medical, and a subset of baccalaureate- and master’s degree–conferring nursing schools were invited to participate in a self-assessment to measure their school’s social mission engagement from January 29 through October 9, 2019. These professions were chosen as representative of the broader range of health professions; future plans for the survey include expanding it to additional fields. Per guidelines from The George Washington University, human participants research approval was not applicable to this study because the survey collected data about schools and programs rather than individuals. The study followed the American Association for Public Opinion Research (AAPOR) reporting guideline for mixed-mode surveys.^6^

We identified US schools using publicly available roster files from the American Dental Education Association for schools granting doctor of dental surgery (DDS) and doctor of medicine in dentistry (DMD) degrees, the American Association of Colleges of Osteopathic Medicine for medical schools granting doctor of osteopathic medicine (DO) degrees, the Liaison Committee on Medical Education for medical schools granting doctor of medicine (MD) degrees, and the American Association of Colleges of Nursing for nursing schools granting baccalaureates and master’s degrees. All US medical schools (203 universities) and dental schools (66 universities) and slightly more than one-half of nursing schools (420 of 781 colleges and universities) were sampled. We limited the number of nursing schools to keep a relative balance between the 3 disciplines. The allocation of nursing schools between baccalaureate- and master’s-level programs was established using a power allocation formula^7^; although master’s-level programs constituted 65% of all US nursing schools, master’s-level schools constituted 57% of the survey sample, which was proportional to the square roots of the frame frequencies for baccalaureate and master’s nursing programs. The sample was stratified based on private vs public status, US Census region (Midwest, Northeast, South, West, Pacific, or Puerto Rico), degree type (DDS, DMD, DO, MD, baccalaureate in nursing, or master’s in nursing), and status as a historically Black college or university (yes or no).

We identified school deans and program directors as the primary target respondents because of their broad insight into their school’s programs and policies and their ability to request data from various internal sources. We also identified secondary target respondents consisting of faculty leaders and diversity and inclusion officers.

### Instrument

The development and testing of the survey used in this study has been previously described^4^; the final instrument is available in eFigure 1 in the Supplement. Survey responses were used to generate numeric scores for 79 indicators (with indicators defined as responses to specific scored questions that indicated the state or level of social mission commitment) across 18 activity areas within 6 domains: (1) educational program (4 activity areas comprising curriculum, extracurricular activities, targeted education, and global health), (2) community engagement (2 activity areas comprising curriculum and community needs and community collaborations), (3) governance (1 activity area comprising school mission), (4) diversity and inclusion (4 activity areas comprising student diversity, faculty diversity, academic leadership diversity, and pathway and pipeline programs), (5) institutional culture and climate (6 activity areas comprising student training, faculty training, student-run clinics, student activism, faculty activism, and primary and community-based care), and (6) research (1 activity area comprising social mission–focused research). A unique survey link was generated to ensure a single final response for each school. We did not collect demographic information from respondents because multiple respondents from an institution could complete different sections of the questionnaire.

The primary unit of measure was the degree-granting program. For medical and dental schools, there was typically 1 primary degree-granting program, so we considered most questions to be relative to the school level. However, nursing schools frequently had several degree-granting programs, with distinct student bodies, curriculum, and, in some cases, leadership. We asked nursing programs that had both undergraduate and graduate programs to choose 1 of their programs on which to report. Based on field testing data, the 30-page survey took a mean (SD) of 6.3 (10.7) hours to complete and required input from a mean (SD) of 5.9 (4.2) people within a school or program.

### Self-assessment Administration

We administered the SMM self-assessment instrument through multiple modes, inviting response to an online survey (Qualtrics) or a paper survey via postal mail. All survey responses from individual schools were strictly confidential and only available to the research team for data analysis purposes. We used multiple strategies to encourage participation, including the creation of an SMM Initiative website,^8^ letters of support from national organizations, webinars, and blog posts. We sent an advance letter announcing the initiative to the dean or program director at each sampled school (with copies to other targeted persons) via both postal mail and email. We subsequently sent an invitation email with an individually tracked link to the online survey instrument, 4 email reminders, and a reminder postcard. We also conducted 2 rounds of reminder phone calls. For those not responding to the initial invitation and the first reminder email within 1 month, we mailed a paper questionnaire that had their school’s name, a unique identification number, and a stamped return envelope. No incentives were offered for survey participation.

### Summary Reports

Each participating school received an individualized confidential summary report approximately 4 months after the survey closed. We converted survey responses into numeric scores and then computed quartiles for area scores and an overall SMM score. Reports showed the school’s quartile position for each area and for social mission overall in relation to other participating schools. This article focuses on aggregate results from the national self-assessment.

### Statistical Analysis

We defined survey completion as returned questionnaires with a 70% or higher completion rate. Survey response rates were calculated using the AAPOR standard response rate 2.^6^ We used pairwise comparison *t* tests with finite population correction and Fisher least significant difference correction (for multiple comparisons) to analyze characteristics of participating and nonparticipating schools. We analyzed the data from 242 schools by conducting descriptive analyses using IBM SPSS Statistics, version 25 (IBM Corp), to create frequency and contingency tables of specific questions within each area. The significance threshold was 2-tailed *P* = .05.

We used pairwise comparison *t* tests with finite population correction and Fisher least significant difference correction to compare the 18 standardized area scores across 3 variables: school discipline, ownership status, and Carnegie Classification of Institutions of Higher Education research classification. To analyze difference in social mission performance by school discipline, we divided schools into 5 groups: (1) dental schools, (2) DO-granting medical schools, (3) MD-granting medical schools, (4) baccalaureate-granting nursing schools, and (5) master’s-granting nursing schools. We did not separate DDS-granting and DMD-granting dental schools given the relatively small sample and the fact that the programs had the same accreditation process and educational requirements.^9^ For ownership status, we classified schools as being either public or private. For comparisons of performance by schools with different Carnegie classifications, we used 4 groupings: (1) doctoral universities with very high research activity (R1), (2) doctoral universities with high (R2) or moderate (R3) research activity, (3) baccalaureate and master’s nursing colleges and universities, and (4) special focus institutions. The special focus designation was based on the concentration of degrees in a single field or set of related fields at both the undergraduate and graduate levels.^10^

## Results

### Survey Response and Sample Characteristics

Of the 689 dental, medical, and nursing schools invited to participate in the self-assessment, 242 schools (35.1%) completed the survey. Of those, 133 (55.0%) were nursing schools, 83 (34.3%) were medical schools, and 26 (10.7%) were dental schools. A total of 51 schools (21.1%) returned their questionnaire on paper. Among nursing schools, 30 (22.6%) reported information about their master’s-level or higher programs, whereas the remainder reported information about their baccalaureate-level programs.

Response rates varied between the 3 disciplines, ranging from 133 of 420 nursing schools (31.7%) to 83 of 203 medical schools (40.9%). These rates may have been impacted by factors including survey length (30 pages), duration (6.3 hours), salience (the importance or current relevance of social mission), and collaboration of multiple respondents (5.9 people).^11,12^ Significant differences in response rates were found across school types, with dental schools (26 of 66 [39.4%]) and medical schools (83 of 203 [40.9%]) more likely to participate than nursing schools (135 of 420 [32.1%]; *P* < .001 for both comparisons). Schools in the West (45 of 103 [43.7%]) were more likely to participate than those in the Midwest (59 of 186 [31.7%]; *P* = .003), Northeast (45 of 133 [33.8%]; *P* = .02), and South (92 of 259 [35.5%]; *P* = .03). Public schools (128 of 329 [38.9%]) were more likely to participate than private schools (114 of 360 [31.7%]; *P* = .003). Doctoral universities with R1 classification (67 of 155 [43.2%]) and R2 or R3 classification (58 of 144 [40.3%]) were more likely to participate than baccalaureate and master’s nursing colleges and universities (69 of 243 [28.4%]; *P* < .001 for comparison with R1 doctoral universities; *P* = .002 for comparison with R2 and R3 doctoral universities) and special focus institutions (48 of 146 [32.9%]; *P* < .001 for comparison with R1 doctoral universities; *P* = .03 for comparison with R2 and R3 doctoral universities) (Table 1).^13^

**Table 1.** Characteristics of Schools Participating in the Social Mission Metrics National Self-assessment in 2019

Characteristic	Reference category	No. (%)		Response rate, %	Contrast estimates^a^					
		Invited	Participated		Reference category 1	*P* value	Reference category 2	*P* value	Reference category 3	*P* value
Total schools, No.	NA	689	242	35.1	NA	NA	NA	NA	NA	NA
Discipline										
Dental	1	66 (9.6)	26 (10.7)	39.4	NA	NA	NA	NA	NA	NA
Medical	2	203 (29.5)	83 (34.3)	40.9	0.015^b^	NA	NA	NA	NA	NA
Nursing	3	420 (61.0)	133 (55.0)	31.7	−0.077	<.001	−0.092	<.001	NA	NA
Region of US^c^										
Midwest	1	186 (27.0)	59 (24.4)	31.7	NA	NA	NA	NA	NA	NA
Northeast	2	133 (19.3)	45 (18.6)	33.8	0.021	.55	NA	NA	NA	NA
South	3	259 (37.6)	92 (38.0)	35.5	0.038	.24	0.017	.61	NA	NA
West	NA	103 (14.9)	45 (18.6)	43.7	0.120	.003	0.099	.02	0.082	.03
Pacific	NA	1 (0.1)	0	0	NA	NA	NA	NA	NA	NA
Puerto Rico	NA	7 (1.0)	1 (0.4)	14.3	NA	NA	NA	NA	NA	NA
Institution type										
Public	1	329 (47.8)	128 (52.9)	38.9	NA	NA	NA	NA	NA	NA
Private	2	360 (52.2)	114 (47.1)	31.7	−0.072	.003	NA	NA	NA	NA
Institution classification^d^^,^^e^										
R1 doctoral university	1	155 (22.5)	67 (27.7)	43.2	NA	NA	NA	NA	NA	NA
R2 or R3 doctoral university	2	144 (20.9)	58 (24.0)	40.3	−0.029	.39	NA	NA	NA	NA
Baccalaureate and master’s nursing college or university	3	243 (35.3)	69 (28.5)	28.4	−0.148	<.001	−0.119	.002	NA	NA
Special focus institution	NA	146 (21.2)	48 (19.8)	32.9	−0.103	<.001	−0.074	.03	0.045	.16

Abbreviation: NA, not applicable.

^a^Results of pairwise *t* tests with finite population correction and Fisher least significant difference correction. The *t *tests were run for each comparison within each category (eg, dental to medical, dental to nursing, medical to nursing).

^b^Significance test not applicable. Full population of dental and medical schools was contacted.

^c^Region is based on US Census regions and divisions.^1^ Pacific and Puerto Rico were not included in the analysis because of the small numbers of insitutions.

^d^Institutional classification is based on the 2018 Carnegie Classification of Institutions of Higher Education; R1 doctoral universities are those with very high research activity, R2 doctoral universities are those with high research activity, and R3 doctoral universities are those with moderate research activity.

^e^Excludes 1 nursing school that did not have a Carnegie classification.

The overall SMM score was able to successfully differentiate schools in terms of their social mission performance. Raw scores ranged from a low of −60.8 points at 1 graduate nursing school to a high of 79.1 points at 1 MD-granting medical school, with 121 schools (50.0%) scoring between −15.2 and 15.4 points. Score cutoffs for the quartiles used in the confidential school reports are shown in eFigure 2 in the Supplement.

### Overall Social Mission Performance

Aggregate results of selected indicators and areas that have been highly prioritized by stakeholders (ie, curriculum, school mission, and curriculum and community needs^5^) are shown in Table 2. Frequencies of all scored indicators by area are available in eTable 1 and eTable 2 in the Supplement.

**Table 2.** Frequency Distribution of Social Mission Factors in Area 1 (Curriculum), Area 5 (School Mission), and Area 6 (Curriculum and Community Needs), 2019

Factor	No./total No. (%)^a^
**Area 1: curriculum**	
Interprofessional education is required for all students^b^	
Yes	200/241 (83.0)
No	41/241 (17.0)
Interprofessional educational setting^c^	
Clinical settings only	10/200 (5.0)
Classroom or simulation settings only	28/200 (14.0)
Both clinical and classroom or simulation settings	162/200 (81.0)
Rotations or courses with underserved patients are available	
Yes	239/242 (98.8)
No	3/242 (1.2)
Rotations or courses with underserved patients are required^c^	
Yes, required of all students	203/239 (84.9)
Yes, required of certain students	15/239 (6.3)
No	21/239 (8.8)
Student participation in rotations or courses with underserved patients (% of students)^c^	
Just a few (<10)	1/35 (2.9)
Some (10-50)	11/35 (31.4)
Most (51-90)	13/35 (37.1)
All or almost all (≥91)	10/35 (28.6)
Rotations or courses with underserved patients are longitudinal^c^	
Yes	199/237 (84.0)
No	38/237 (16.0)
Social determinants of health are included in curriculum^b^	
Yes, in required courses	233/242 (96.3)
Yes, in elective courses	4/242 (1.7)
No	5/242 (2.1)
Social determinants of health are integrated across multiple years of study^c^	
Yes, across all years of study	116/235 (49.4)
Yes, across multiple years of study	95/235 (40.4)
No	24/235 (10.2)
Health disparities are included in curriculum^b^	
Yes, in required courses	232/242 (95.9)
Yes, in elective courses	6/242 (2.5)
No	4/242 (1.7)
Health disparities are integrated across multiple years of study^c^	
Yes, across all years of study	118/235 (50.2)
Yes, across multiple years of study	96/235 (40.9)
No	21/235 (8.9)
LGBTQ health is included in curriculum^b^	
Yes, in required courses	170/242 (70.2)
Yes, in elective courses	13/242 (5.4)
No	59/242 (24.4)
LGBTQ health is integrated across multiple years of study^c^	
Yes, across all years of study	39/183 (21.3)
Yes, across multiple years of study	99/183 (54.1)
No	45/183 (24.6)
**Area 5: school mission**	
Written mission statement^b^	
Yes	240/241 (99.6)
No	1/241 (0.4)
Terms mentioned in mission statement^c^	
Social determinants of health	24/169 (14.2)^d^
Underserved, underrepresented, and/or disadvantaged populations	82/169 (48.5)^d^
Health equity or health disparities	63/169 (37.3)^d^
A specific community of commitment^c^	80/236 (33.9)^d^
Current strategic plan^b^	
Yes	215/240 (89.6)^d^
No	25/240 (10.4)^d^
Terms mentioned in strategic plan^c^	
Social determinants of health	36/217 (16.6)^d^
Underserved, underrepresented, and/or disadvantaged populations	112/217 (51.6)^d^
Health equity or health disparities	69/217 (31.8)^d^
A specific community of commitment	99/211 (46.9)^d^
**Area 6: curriculum and community needs**	
School or affiliated hospital or institution conducted a formal or informal CHNA in past 5 y^b^	
Yes, formal	107/187 (57.2)
Yes, informal	46/187 (24.6)
Neither	34/187 (18.2)
Design of curriculum is explicitly informed by CHNA^c^	
Substantially	29/144 (20.1)
Moderately	42/144 (29.2)
Slightly	61/144 (42.4)
Not at all	12/144 (8.3)

Abbreviations: CHNA, community health needs assessment; LGBTQ, lesbian, gay, bisexual, transgender, and queer or questioning.

^a^ Nonrespondents and missing data are not included in the denominator.

^b^ Contingency question.

^c^ Response based on contingency question.

^d^ Respondents could select multiple items inclusive of social determinants of health; underserved, underrepresented, and/or disadvantaged populations; health equity or health disparities; or community of commitment.

#### Curriculum (Area 1)

Within the curriculum area, almost all schools (239 of 242 [98.8%]) offered rotations or courses in which students could work with underserved patients, with 203 of 239 schools (84.9%) requiring these courses of all students and 199 of 237 schools (84.0%) reporting that courses or rotations were longitudinal. Most schools included social determinants of health in either their required courses (233 of 242 [96.3%]) or elective courses (4 of 242 schools [1.7%]), although only 116 schools (49.4%) integrated social determinants of health across all years of study. Most schools also included health disparities in either their required courses (232 of 242 [95.9%]) or elective courses (6 of 242 [2.5%]); however, only 118 of 235 schools (50.2%) integrated health disparities across all years of study.

#### School Mission (Area 5)

Almost every school had a written mission statement (240 of 241 [99.6%]) and current strategic plan (215 of 240 [89.6%]). A total of 110 of 240 schools (45.8%) reported the presence of at least 1 of 3 important social mission terms in their mission statement, with the mission statements of 24 of 169 schools (14.2%) mentioning social determinants of health; 82 of 169 schools (48.5%) mentioning underserved, underrepresented, and/or disadvantaged populations; and 63 of 169 schools (37.3%) mentioning health equity or health disparities. Overall, 126 of 215 schools (58.6%) reported the presence of 1 of the social mission terms in their strategic plan, with 36 of 217 schools (16.6%) mentioning social determinants of health; 112 of 217 schools (51.6%) mentioning underserved, underrepresented, and/or disadvantaged populations; and 69 of 217 schools (31.8%) mentioning health equity or health disparities. Only 80 of 236 schools (33.9%) identified a specific community of commitment (defined as a medically or socially underserved community, which could be an underserved geographic area [local or regional], demographic group, or category of patient that the school had explicitly targeted as a focus for their work^5^) in their mission statement, and only 99 of 211 schools (46.9%) included a specific community of commitment in their strategic plan.

#### Curriculum and Community Needs (Area 6)

Most schools (153 of 187 [81.8%]) had conducted either a formal (107 schools) or informal (46 schools) community health needs assessment within the past 5 years. A total of 71 of 144 schools (49.3%) substantially or moderately used the community health needs assessment to inform their curriculum.

### Comparative Analyses

#### Cross-disciplinary Comparisons

**Figure 1.**
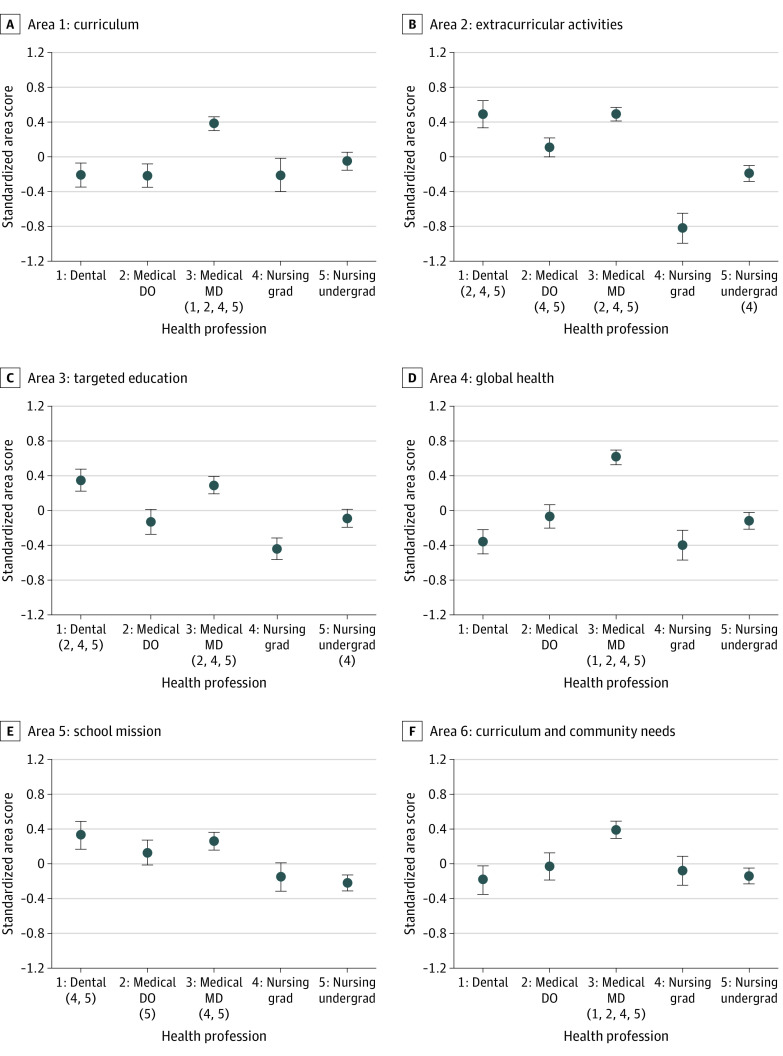
Standardized Area Scores by Health Professions Discipline From the Social Mission Metrics National Self-assessment for Areas 1 to 6, 2019 Group mean standardized area scores of the groups (numbered 1 through 5 from left to right) were derived using pairwise *t* tests with finite population correction and Fisher least significant difference correction. Numbers in parentheses under a group indicate the groups with mean scores significantly lower than those in that group. A total of 26 dental schools, 25 DO-granting, 58 MD-granting medical schools, 31 master’s-granting nursing schools, and 102 undergraduate nursing schools were included in the analysis. Whiskers represent SEs. DO indicates doctor of osteopathic medicine; grad, graduate-level programs; MD, doctor of medicine; and undergrad, undergraduate-level programs.

**Figure 2.**
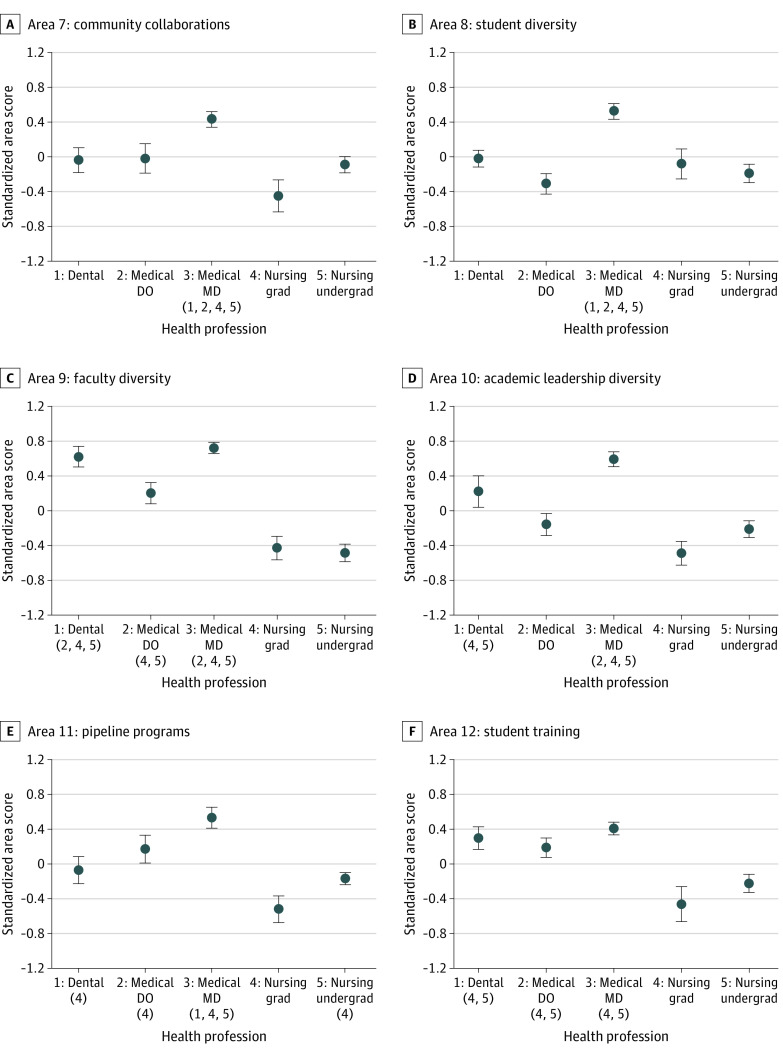
Standardized Area Scores by Health Professions Discipline from the Social Mission Metrics National Self-assessment for Areas 7 to 12, 2019 Group mean standardized area scores of the groups (numbered 1 through 5 from left to right) were derived using pairwise *t* tests with finite population correction and Fisher least significant difference correction. Numbers in parentheses under a group indicate the groups with mean scores significantly lower than those in that group. A total of 26 dental schools, 25 DO-granting, 58 MD-granting medical schools, 31 master’s-granting nursing schools, and 102 undergraduate nursing schools were included in the analysis. Whiskers represent SEs. DO indicates doctor of osteopathic medicine; grad, graduate-level programs; MD, doctor of medicine; and undergrad, undergraduate-level programs.

When comparing social mission performance across 5 groups of health professions, we identified significant differences between specific pairs of disciplines for every area, with the exception of area 18 (Figure 1 and Figure 2; eFigure 3 in the Supplement). For example, in area 1 (curriculum), MD-granting medical schools had a mean (SE) standardized area score of 0.38 (0.08) points, which was significantly higher than the standardized area scores of dental schools (mean [SE], −0.21 [0.14] points; *P* < .001), DO-granting medical schools (mean [SE], −0.22 [0.13] points; *P* < .001), graduate nursing schools (mean [SE], −0.21 [0.19] points; *P* = .005), and undergraduate nursing schools (mean [SE], −0.05 [0.10] points; *P* = .001). Medical schools granting MD degrees scored significantly higher (mean [SE], 0.52 [0.12] points) than dental schools (mean [SE], −0.08 [0.15] points; *P* = .002), graduate nursing schools (mean [SE], −0.52 [0.15]; *P* < .001), and undergraduate nursing schools (mean [SE], −0.17 [0.07]; *P* < .001) in area 11 (pathway and pipeline programs).

#### Public vs Private Institutions

**Figure 3.**
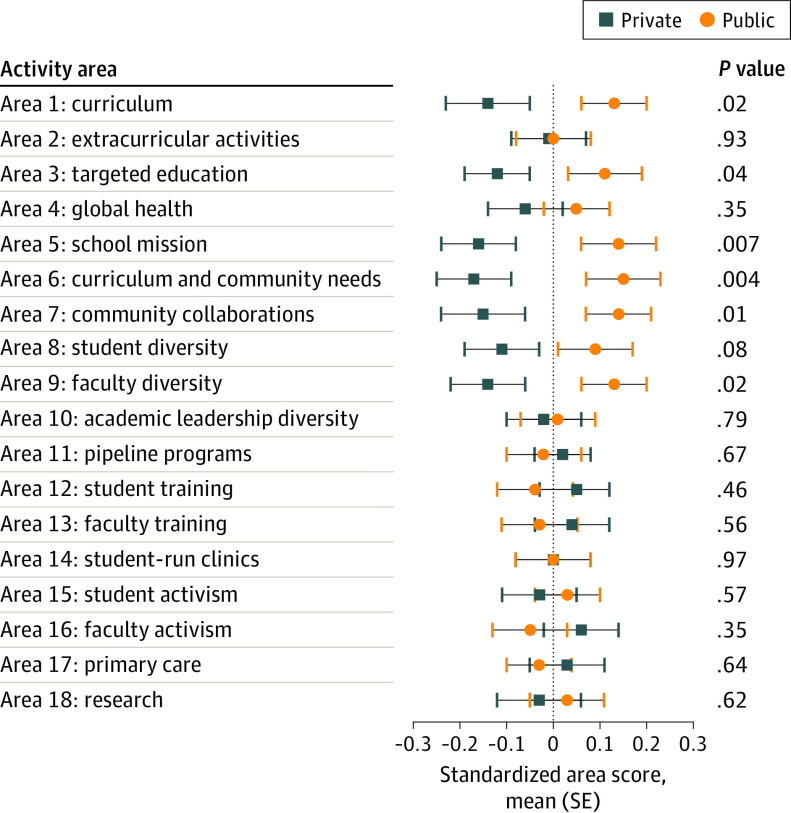
Social Mission Metrics National Self-assessment Performance Activity Areas by Institution Type and Statistical Significance, 2019 The significance threshold was 2-tailed *P* = .05.

Public schools performed significantly better than private schools in area 1 (curriculum; mean [SE] standardized area score, 0.13 [0.07] points vs −0.14 [0.09] points, respectively; *P* = .02), area 3 (targeted education; mean [SE] standardized area score, 0.11 [0.08] points vs −0.12 [0.07] points; *P* = .04), area 5 (school mission; mean [SE] standardized area score, 0.14 [0.08] points vs −0.16 [0.08] points; *P* = .007), area 6 (curriculum and community needs; mean [SE] standardized area score, 0.15 [0.08] points vs −0.17 [0.08] points; *P* = .004), area 7 (community collaborations; mean [SE] standardized area score, 0.14 [0.07] points vs −0.15 [0.09] points; *P* = .01), and area 9 (faculty diversity; mean [SE] standardized area score, 0.13 [0.07] points vs −0.14 [0.08] points; *P* = .02). No significant difference was found between public and private school performance in the remaining areas (Figure 3).

#### Carnegie Classification

When assessing schools by Carnegie research classification group, schools or programs at R1 doctoral universities and special focus institutions performed better than the other categories in several areas. For example, in area 2 (extracurricular activities), the mean (SE) standardized area score was 0.25 (0.09) points for R1 doctoral universities and 0.20 (0.12) points for special focus institutions vs −0.05 (0.12) points for R2 and R3 doctoral universities (*P* = .06 for comparison with R1 doctoral universities and *P* = .13 for comparison with special focus institutions) and – 0.30 (0.12) points for baccalaureate and master’s nursing colleges and universities (*P* < .001 for comparison with R1 doctoral universities and *P* = .003 for comparison with special focus institutions) (eFigure 4 in the Supplement). There was a notable exception for area 17 (primary care), in which R1 doctoral universities (mean [SE] standardized area score, −0.41 [0.08] points) performed significantly worse than R2 and R3 doctoral universities (mean [SE] standardized area score, 0.22 [0.12] points; *P* < .001), baccalaureate and master’s nursing colleges and universities (mean [SE] standardized area score, 0 [0.12] points; *P* = .005), and special focus institutions (mean [SE] standardized area score, 0.33 [0.13] points; *P* < .001). No significant differences between any groups were found in area 5 (school mission), area 13 (faculty training), area 16 (faculty activism), and area 18 (research focused on social mission).

## Discussion

This survey study found that it was possible to measure a wide range of social mission–advancing activities with an interdisciplinary approach on a national scale. The participation of more than 240 schools, despite the substantial time commitment at the senior leadership level, revealed that schools were eager to measure their social mission commitment and understand their relative performance compared with other institutions.

Our results highlighted many positive findings about the state of social mission commitment in health professions schools. For example, almost all participating schools included important social mission content areas, such as social determinants of health and health disparities, in their curriculum. However, there is still progress to be made; it was less common for these content areas to be integrated across all years of study, despite recommendations to teach this topic within a longitudinal framework.^14^ There was also substantial room for improvement in explicitly aligning curriculum with local community needs. The Association of American Medical Colleges has advocated for the incorporation of a local community health needs assessment into the curriculum at academic health care centers.^15^ However, only 49.3% of schools with access to such a tool reported that it moderately or substantially informed their curriculum. Health professions schools are not yet rising to the challenge of ensuring that graduates are well prepared for clinical practice, regardless of discipline, while acknowledging and enhancing their role in improving local community health.

Although academic health disciplines are diverse and distinct from one another in function, history, and training, all share a common goal of improving health. This study’s intent was to bring awareness and create synergies across the school types in the pursuit of social mission engagement and performance. Patterns were observable in the data that may be beneficial for various stakeholders to note. For example, the better social mission performance observed in many areas among public schools, R1 doctoral universities, and special focus institutions warrants further investigation into factors associated with those differences. Certain differences between disciplines may be at least partially explainable by external pressures. For example, it was not unexpected that medical schools scored higher than dental and nursing schools in area 11 (pathway and pipeline programs) given that the Liaison Committee on Medical Education is the only major accrediting institution across the 3 disciplines assessed that specifically includes pipeline programs in its standards.^16^ Accreditation may be a useful incentive to further the widespread advancement of social mission in health professions schools.^16,17,18,19,20^ Ongoing analyses that examine the association of survey data with institutional characteristics and external outcome measures may elucidate additional reasons for these patterns and identify mechanisms for further transforming health professions education to meet societal needs.

### Limitations

This study has several limitations. Nonresponse bias is a concern in any survey; we may have received more responses from schools that emphasize and value social mission than those that do not. Although participating schools were well distributed across the 4 institutional classifications, research-intensive schools were more likely to respond across the 3 disciplines, perhaps because they have greater administrative capacity or financial resources, enabling them to focus on social mission work. However, the size of the sample and the wide range of SMM scores suggest that the sample includes a diversity of social mission performance levels.

Social mission can manifest in myriad ways; thus, some individual school programs and policies that have a role in health equity may not be measured by our survey. Through our survey development and testing process, we tried to meet that challenge by focusing on universally important criteria, although schools may go beyond these criteria and have different areas of emphasis. In addition, a quantitative measurement tool is unable to capture the full scope of differences in the quality and depth of social mission activities. Two schools may provide identical responses to a given set of factors, but what they are doing in practice may differ substantially.

Several limitations are inherent when using self-reported data. Despite assurances that individual survey responses were confidential and strictly for the purposes of self-assessment, social desirability bias remains a concern. We targeted our survey to school leaders, but we may have received different responses if querying other stakeholders, such as students, staff, or community members. Robust internal and external validations of the survey instrument based on the collected data are ongoing.

Additional planned work will focus on understanding the impact of participation in the self-assessment for schools and developing accompanying tools to help schools create actionable plans based on self-assessment results. We aim to repeat the survey every 3 to 5 years to allow schools to measure progress over time, a step that will be particularly interesting given the consequences of the COVID-19 pandemic and renewed calls for racial justice in recent years. Researchers and policy makers can use the SMM framework based on 18 activity areas when a comprehensive view of social mission is warranted.^16^

## Conclusions

The results of this survey study highlight the fact that social mission is an important aspect of health professions education, especially in the current historic moment as institutions seek to advance their health equity and social justice commitments. To motivate systemic change in pursuit of health equity, it is important to measure the current state and future progress of social mission in health professions education. The results of the national self-assessment reported in this study suggest both the feasibility and utility of doing so.

## Notes

Supplement. **eTable 1.** Frequency Distribution of Social Mission Indicators in Areas 2 to 4 and Areas 7 to 18, 2019 **eTable 2.** Descriptive Statistics for Social Mission Indicators in Areas 8 to 11, 2019 **eFigure 1.** Social Mission Metrics Self-assessment Survey for Medical Schools, 2019 **eFigure 2.** Frequency Histogram of Participating Self-assessment Dental, Medical, and Nursing Schools’ Overall Social Mission Score, 2019 **eFigure 3.** Standardized Area Scores by Health Professions Discipline From the Social Mission Metrics National Self-assessment for Areas 13 to 18, 2019 **eFigure 4.** Standardized Area Scores by Carnegie Classification From the Social Mission Metrics National Self-assessment for Areas 1 to 18, 2019
