# Supplementary material for: Analysis of Social Mission Commitment at Dental, Medical, and Nursing Schools in the US
Source: JAMA Netw Open. 2022 May 9;5(5):e2210900. doi: 10.1001/jamanetworkopen.2022.10900 (PMC9086841; doi:10.1001/jamanetworkopen.2022.10900)
Supplement: Supplement. — eTable 1. Frequency Distribution of Social Mission Indicators in Areas 2 to 4 and Areas 7 to 18, 2019 eTable 2. Descriptive Statistics for Social Mission Indicators in Areas 8 to 11, 2019 eFigure 1. Social Mission Metrics Self-assessment Survey for Medical Schools, 2019 eFigure 2. Frequency Histogram of Participating Self-assessment Dental, Medical, and Nursing Schools’ Overall Social Mission Score, 2019 eFigure 3. Standardized Area Scores by Health Professions Discipline From the Social Mission Metrics National Self-assessment for Areas 13 to 18, 2019 eFigure 4. Standardized Area Scores by Carnegie Classification From the Social Mission Metrics National Self-assessment for Areas 1 to 18, 2019 [file jamanetwopen-e2210900-s001.pdf]

Supplementary Online Content

Batra S, Orban J, Zhang H, et al. Analysis of social mission commitment at dental, medical, and nursing schools in the US. *JAMA Netw Open*. 2022;5(5):e2210900. doi:10.1001/jamanetworkopen.2022.10900

eTable 1. Frequency Distribution of Social Mission Indicators in Areas 2 to 4 and Areas 7 to 18, 2019

eTable 2. Descriptive Statistics for Social Mission Indicators in Areas 8 to 11, 2019

eFigure 1. Social Mission Metrics Self-assessment Survey for Medical Schools, 2019

eFigure 2. Frequency Histogram of Participating Self-assessment Dental, Medical, and Nursing Schools' Overall Social Mission Score, 2019

eFigure 3. Standardized Area Scores by Health Professions Discipline From the Social Mission Metrics National Self-assessment for Areas 13 to 18, 2019

eFigure 4. Standardized Area Scores by Carnegie Classification From the Social Mission Metrics National Self-assessment for Areas 1 to 18, 2019

This supplementary material has been provided by the authors to give readers additional information about their work.

© 2022 Batra S et al. *JAMA Network Open*.

eTable 1. Frequency Distribution of Social Mission Indicators in Areas 2 to 4 and Areas 7 to 18, 2019

Indicator	No. (%)[a]
**Area 2: Extracurricular Activities**	
Service-learning or volunteer hours requirement for graduation[b]	
Yes, required of all students	100/241 (41.5)
Yes, required of some students	11/241 (4.6)
No	130/241 (53.9)
If not required, percent of students who volunteer or participate in service-learning[c]	
All or nearly all (approx. 91% or more)	24/117 (20.5)
Most (51-90%)	47/117 (40.2)
Some (11-50%)	36/117 (30.8)
Just a few (10% or less)	10/117 (8.5)
If required, total service-learning or volunteer hour time commitment[c]	
No specific time commitment	12/112 (10.7)
1-10 hours	18/112 (16.1)
11-30 hours	35/112 (31.3)
31-50 hours	12/112 (10.7)
>50 hours	35/112 (31.3)
Extracurricular activities addressing health disparities/social determinants of health available[b]	
Yes	195/233 (83.7)
No	38/233 (16.3)
Extracurricular activities - Percent of students who participate[c]	
All or nearly all (91% or more)	51/186 (27.4)
Most (51-90%)	53/186 (28.5)
Some (10-50%)	72/186 (38.7)
Just a few (less than 10%)	10/186 (5.4)
**Area 3: Targeted Education**	
School/institution offers a certification or degree in Public Health[b]	
Yes	118/241 (49.0)
No	118/241 (51.0)
Public Health certification or degree – Percent completed over last 4 graduation classes	
All or nearly all (91% or more)	7/100 (7.0)
Most (51-90%)	1/100 (1.0)
Some (10-50%)	9/100 (9.0)
Just a few (less than 10%)	53/100 (53.0)
None	30/100 (30.0)
Medical schools only: Education programs within the school/institution that train	
Community Health Workers	14/78 (17.9)
Medical Assistants	6/79 (7.6)

© 2022 Batra S et al. *JAMA Network Open*.

Dental schools only: Education programs within the school/institution that train	
Dental Hygienists	9/26 (34.6)
Dental Assistants	2/25 (8.0)
Nursing schools only: Education programs within the school/institution that train	
Community Health Workers	29/124 (23.4)
Nurse's Aides	8/130 (6.2)
**Area 4: Global Health**	
Formal partnership with an institution in a low or middle income country (LMIC)[b]	
Yes	121/232 (52.2)
No	111/232 (47.8)
Facilitate students from LMIC to train or study at your institution[c]	
Yes	59/111 (53.2)
No	52/111 (46.8)
Global health rotations in LMIC available[b]	
Yes	145/238 (60.9)
No	93/238 (39.1)
Global health rotations in LMIC – percent of students who participate[c]	
All or nearly all (91% or more)	3/140 (2.1)
Most (51-90%)	3/140 (2.1)
Some (10-50%)	76/140 (54.3)
Just a few (less than 10%)	58/140 (41.4)
Global health rotations in LMIC – average duration[c]	
<2 weeks	39/145 (26.9)
2-4 weeks	96/145 (66.2)
>4 weeks	10/145 (6.9)
**Area 7: Community Collaborations**	
Clinics, offices, or programs in off-campus local community[b]	
Yes	231/238 (97.1)
No	7/238 (2.9)
Clinics in community – Compensation for devoted faculty and staff	
Yes	100/220 (45.5)
No	120/220 (54.5)
Feedback from the community to the school about these programs	
Yes	189/207 (91.3)
No	18/207 (8.7)
Formal or established collaborations with	
Legal professionals	79/215 (36.7)
Churches, temples or other faith-based organizations	158/226 (69.9)
Federally Qualified Health Center(s)	193/226 (85.4)
Local K-12 schools	206/235 (87.7)

© 2022 Batra S et al. *JAMA Network Open*.

Local community colleges	147/230 (63.9)
Local health department	204/232 (87.9)
Philanthropic organizations	173/228 (75.9)
**Area 8: Student Diversity (see following table for additional indicators in this area)**	
Admissions policy holistic review of applicants	
Yes, as a matter of written policy	142/232 (61.2)
Yes, as a matter of informal practice	55/232 (23.7)
No	35/232 (15.1)
Student's sexual orientation and/or gender identity data collected	
Yes	47/242 (19.7)
No	191/242 (80.3)
**Area 9: Faculty diversity[d] (see following table for additional indicators in this area)**	
Faculty openly identify as LGBTQ	
None	56/151 (37.1)
1-2	49/151 (32.5)
3-4	17/151 (11.3)
5-9	7/151 (4.6)
10 or more	22/151 (14.6)
**Area 10: Academic leadership diversity (see following table for additional indicators in this area)**	
Academic leader(s) openly LGBTQ	
Yes	57/145 (39.3)
No	88/145 (60.7)
**Area 11: Pipeline programs (see following table for additional indicators in this area)[d]**	
Sponsoring, running or assisting with any pipeline programs targeted to K-12 students[b]	
Yes	146/232 (62.9)
No	86/232 (37.1)
Sponsoring, running or assisting with any pipeline programs targeted to undergraduate college students[b]	
Yes	130/221 (58.8)
No	91/221 (41.2)
**Area 12: Student training**	
Students receive training in unconscious/implicit bias	
All or nearly all (91% or more)	102/183 (55.7)
Most (51-90%)	27/183 (14.8)
Some (10-50%)	31/183 (16.9)
Just a few (less than 10%)	13/183 (7.1)
Not offered	10/183 (5.5)
Students receive training in cultural competency or cultural humility	
All or nearly all (91% or more)	149/198 (75.3)
Most (51-90%)	29/198 (14.6)

© 2022 Batra S et al. *JAMA Network Open*.

Some (10-50%)	14/198 (7.1)
Just a few (less than 10%)	4/198 (2.0)
Not offered	2/198 (1.0)
Students receive training in advocacy on issues related to health	
All or nearly all (91% or more)	133/196 (67.9)
Most (51-90%)	32/196 (16.3)
Some (10-50%)	21/196 (10.7)
Just a few (less than 10%)	5/196 (2.6)
Not offered	5/196 (2.6)
Students receive training in social determinants of health	
All or nearly all (91% or more)	151/197 (76.6)
Most (51-90%)	31/197 (15.7)
Some (10-50%)	8/197 (4.1)
Just a few (less than 10%)	3/197 (1.5)
Not offered	4/197 (2.0)
**Area 13: Faculty training**	
Faculty receive training in unconscious/implicit bias	
All or nearly all (91% or more)	62/195 (31.8)
Most (51-90%)	48/195 (24.6)
Some (10-50%)	61/195 (31.3)
Just a few (less than 10%)	14/195 (7.2)
Not offered	10/195 (5.1)
Faculty receive training in cultural competency or cultural humility	
All or nearly all (91% or more)	72/193 (37.3)
Most (51-90%)	54/193 (28.0)
Some (10-50%)	53/193 (27.5)
Just a few (less than 10%)	6/193 (3.1)
Not offered	8/193 (4.1)
Faculty receive training in advocacy on issues related to health	
All or nearly all (91% or more)	56/183 (30.6)
Most (51-90%)	42/183 (23.0)
Some (10-50%)	53/183 (29.0)
Just a few (less than 10%)	19/183 (10.4)
Not offered	13/183 (7.1)
Faculty receive training in social determinants of health	
All or nearly all (91% or more)	51/183 (27.9)
Most (51-90%)	57/183 (31.1)
Some (10-50%)	49/183 (26.8)
Just a few (less than 10%)	15/183 (8.2)

© 2022 Batra S et al. *JAMA Network Open*.

Not offered	11/183 (6.0)
**Area 14: Student-run clinics**	
Students volunteer in student-run clinics in the past three years[b]	
Yes	121/226 (53.5)
No	105/226 (46.5)
Percent of students who volunteer at student-run health clinics[c]	
All or nearly all (91% or more)	30/120 (25.0)
Most (51-90%)	30/120 (25.0)
Some (10-50%)	44/120 (36.7)
Just a few (less than 10%)	13/120 (10.8)
None	3/120 (2.5)
Percent of faculty or staff members who actively give time to student-run health clinics[c]	
All or nearly all (91% or more)	1/120 (0.8)
Most (51-90%)	3/120 (2.5)
Some (10-50%)	47/120 (39.2)
Just a few (less than 10%)	66/120 (55.0)
None	3/120 (2.5)
School provides compensation for faculty and staff time devoted to student-run clinics[c]	
Yes	44/117 (37.6)
No	73/117 (62.4)
**Area 15: Student activism**	
Students actively involved in community-based programs addressing health disparities or social determinants	
All or nearly all (91% or more)	66/196 (33.7)
Most (51-90%)	51/196 (26.0)
Some (10-50%)	60/196 (30.6)
Just a few (less than 10%)	15/196 (7.7)
None	4/196 (2.0)
Financial support for community-oriented student activities outside of the curriculum	
Yes	105/211 (49.8)
No	106/211 (50.2)
Underrepresented students active in minority professional associations	
All or nearly all (91% or more)	34/149 (22.8)
Most (51-90%)	39/149 (26.2)
Some (10-50%)	46/149 (30.9)
Just a few (less than 10%)	28/149 (18.8)
None	2/149 (1.3)
**Area 16: Faculty activism**	
Faculty actively involved in community-based programs addressing health disparities or social determinants	

© 2022 Batra S et al. *JAMA Network Open*.

All or nearly all (91% or more)	13/188 (6.9)
Most (51-90%)	40/188 (21.3)
Some (10-50%)	105/188 (55.9)
Just a few (less than 10%)	30/188 (16.0)
Faculty advocating for change in health issues	
All or nearly all (91% or more)	13/185 (7.0)
Most (51-90%)	41/185 (22.2)
Some (10-50%)	91/185 (49.2)
Just a few (less than 10%)	40/185 (21.6)
School recognizes or rewards faculty participation in community-based programs and advocacy activities[b]	
Yes	134/206 (65.0)
No	72/206 (35.0)
School recognizes or reward faculty - method[c]	
Promotion criteria explicitly recognize these contributions	86/183 (47.0)
Awards for community activities or advocacy	67/183 (36.6)
Compensation/pay raise	13/7.1 (7.1)
Other	17/183 (9.3)
**Area 17: Primary care[d]**	
Encouragement from school to enter primary care or general community-based practice	
A great deal of encouragement	106/236 (44.9)
Some encouragement	68/236 (28.8)
A little encouragement	13/236 (5.5)
Neither encourage or discourage	48/236 (20.3)
A great deal of discouragement	1/236 (0.4)
**Area 18: Research**	
Number of community engaged research projects	
None	47/198 (23.7)
One	30/198 (15.2)
2 or 3	45/198 (22.7)
4 to 9	34/198 (17.2)
10 or more	42/198 (21.2)
Number of health equity or health disparity research projects	
None	50/198 (25.3)
One	29/198 (14.6)
2 or 3	44/198 (22.2)
4 to 9	31/198 (15.7)
10 or more	44/198 (22.2)
Number of health promotion or disease prevention research projects	
None	30/202 (14.9)

© 2022 Batra S et al. *JAMA Network Open*.

One	28/202 (13.9)
2 or 3	53/202 (26.2)
4 to 9	34/202 (16.8)
10 or more	57/202 (28.2
Number of research into social determinants of health projects	
None	59/190 (31.1)
One	33/190 (17.4)
2 or 3	33/190 (17.4)
4 to 9	25/190 (13.2)
10 or more	40/190 (21.1)
Number of health or research needs assessment in community projects	
None	53/186 (28.5)
One	36/186 (19.4)
2 or 3	43/186 (23.1)
4 to 9	27/186 (14.5)
10 or more	27/186 (14.5)
Percent of total research portfolio focused in all the above categories combined	
0-10%	91/218 (41.7)
11-25%	47/218 (21.6)
26-50%	36/218 (16.5)
> 50%	44/218 (20.2)
Community Health Needs Assessment (CHNA) influence on research	
Substantially	18/172 (10.5)
Moderately	45/172 (26.2)
Slightly	52/172 (30.2)
Not at all	15/172 (8.7)
Our school doesn't have a CHNA	42/172 (24.4)

[a] Nonrespondents and missing data are not included in the denominator
[b] Contingency question
[c] Response based on contingency question
[d] Additional open-text indicators for the Area can be located in Supplemental Digital Appendix 5
[e] Multi-select response inclusive of social determinants of health, underserved/underrepresented/disadvantaged populations, health equity or health disparities, or community of commitment.

eTable 2. Descriptive Statistics for Social Mission Indicators in Areas 8 to 11, 2019

Indicator	Mean % (SD)	No. of Schools Responding
Student self-reported race/ethnicity		
Hispanic or Latinx	8.7 (11.6)	242
American Indian or Alaskan Native (not Hispanic)	0.5 (1.0)	241
Asian/Pacific Islander (not Hispanic)	12.8 (12.6)	242
Black or African American (not Hispanic)	8.5 (13.6)	242
Native Hawaiian or Other Pacific Islander	0.4 (1.5)	241
White (not Hispanic)	56.5 (25.7)	241
Two or more races (not Hispanic)	2.8 (3.7)	241
Not reported/not known	3.1 (6.8)	240
International student (nonresident alien)	1.0 (2.7)	240
Graduation rate by race/ethnicity	85.5 (21.9)	231
Hispanic or Latinx	78.1 (34.6)	145
American Indian or Alaskan Native (not Hispanic)	110 (66.2)	110
Asian/Pacific Islander (not Hispanic)	77.3 (34.1)	137
Black or African American (not Hispanic)	77.0 (31.2)	144
White (not Hispanic)	86.7 (23.1)	152
Two or more races, other race	69.6 (41.6)	115
Race not reported	69.6 (39.6)	117
Students from the community of commitment	55.6 (33.1)	61
First generation college students	27.7 (20.7)	91
Graduation rate for 1st generation college students	87.5 (18.3)	48
Scholarship and grant dollars that are need-based	66.0 (32.7)	126
Faculty self-reported race/ethnicity		
Hispanic or Latinx	3.1 (6.2)	241
American Indian or Alaskan Native (not Hispanic)	0.3 (1.6)	241
Asian/Pacific Islander (not Hispanic)	7.8 (9.8)	241
Black or African American (not Hispanic)	6.6 (13.6)	241
Native Hawaiian or Other Pacific Islander	0.2 (1.7)	241
White (not Hispanic)	60.0 (33.9)	240
Two or more races (not Hispanic)	1.1 (6.7)	241
Not reported/not known	2.1 (6.4)	241
International employee (Nonresident alien)	0.5 (2.0)	241
Faculty who are women	69.4 (27.1)	229
Academic leader self-reported race/ethnicity		
Hispanic or Latinx	2.9 (9.3)	240
American Indian or Alaskan Native (not Hispanic)	0.3 (2.4)	240

© 2022 Batra S et al. *JAMA Network Open*.

Asian (not Hispanic)	4.2 (8.6)	239
Black or African American (not Hispanic)	5.7 (16.0)	239
Native Hawaiian or Other Pacific Islander	0.6 (5.3)	240
White (not Hispanic)	68.3 (37.6)	239
Two or more races (not Hispanic)	0.4 (2.5)	240
Not reported/not known	0.5 (3.1)	240
International employee (Nonresident alien)	0.02 (0.4)	240
Academic leaders who are women	66.7 (32.7)	219
Number of K-12 students impacted annually[a]	386.2 (1191.7)	100
Participating K-12 students from underrepresented racial/ethnic minority groups[a]	75.9 (24.0)	81
Participating K-12 students in free or reduced lunch[a]	78.9 (23.1)	42
Undergraduate students impacted annually[a]	119.0 (228.5)	81
Participating undergraduate students from underrepresented racial/ethnic minority groups[a]	70.9 (28.3)	70
Participating undergraduate students who are 1st generation to go to college[a]	56.1 (26.0)	44
Participating undergraduate students who enter health profession education[a]	69.1 (27.9)	45
Medical graduates entering residency in family medicine	16.5 (13.2)	67
Dental graduates entering general practice dentistry	76.3 (15.7)	16
Nursing graduates entering public health nursing or work with underserved populations	32.6 (33.4)	41
Medical graduates entering primary care	46.1 (18.0)	47
Nurse Practitioner graduates entering primary care positions	82.8 (13.2)	20

[a] Response based on contingency question from eTable 1

© 2022 Batra S et al. *JAMA Network Open*.

eFigure 1. Social Mission Metrics Self-Assessment Survey for Medical Schools, 2019

SOCIAL MISSION METRICS QUESTIONNAIRE

Self-Assessment Tool

INTRODUCTION AND INSTRUCTIONS

Thank you for participating in the self-assessment process by filling out this questionnaire about your school. This questionnaire aims to assess the social mission of your school, defined as the contribution of the school in its mission, programs, and the performance of its graduates, faculty and leadership to addressing the health disparities of the society in which it exists.

Background

The Social Mission Self-Assessment aims to measure the social mission engagement of a health professions school, track it over time, and compare it to national norms. The questionnaire is based on pre-existing tools to assess aspects of social mission, literature review, and expert opinion. It was created with the guidance of a national multidisciplinary Advisory Committee. It has been field tested at 65 dental, medical, and nursing schools and revised based on this fielding process.

Who will participate?

All dental and medical school deans in the United States are invited to participate in the self-assessment process. Given the large number of nursing schools in the U.S., compared to the other disciplines, a randomly selected sample of 400 nursing school deans will also get this invitation. Nursing schools that were not randomly selected to receive an invitation to participate may elect to participate by contacting the study team.

What will be done with the results?

The research team will provide a <u>confidential</u> summary report to the primary respondent at the school. This will include a summary of the responses of your school compared to aggregated data of all participating schools within your discipline. The summary report may be used to further engage in self-analysis and reflection of their baseline performance to establish performance-improving changes.

How will the confidentiality of my answers be protected?

Your responses will only be shared with the study team. You will NOT receive any information on the responses of other schools, and no other schools will receive information on the responses of your school. No information on a particular school's standing will be shared or published without <u>explicit</u> consent from that school.

Who should answer these questions?

We've sent this survey to you as someone who has insight into your school's programs and is in a position to request data from various internal sources. We don't expect that you'll have all the answers at your fingertips, so we encourage you to look at databases at your institution and to consult with people in other departments or units who might have some of the information requested. You may delegate a primary respondent other than yourself to take responsibility for aggregating the required information.

In field tests of the survey, the following departments were typically consulted by the primary participant:

© 2022 Batra S et al. *JAMA Network Open*.

- Academic Affairs
- Curricular/Educational Affairs
- Student Affairs
- Diversity and Inclusion
- Faculty Affairs
- Admissions
- Financial Aid

What if I don't know the answer?

Please select the answer choice "Don't Know/Information Not Available" or leave the question blank if the "Don't Know" option is not there.

What if I don't wish to share an answer?

Participation is voluntary and for your own self-reflection, so we encourage schools to answer all questions if possible. If there is any requested information that you or your school does not wish to share, please leave the item blank or note that you decline to answer that item. If you are willing, we would be interested in understanding why you may not be interested in sharing the information as it will assist us in better refining the questionnaire.

How will participating be beneficial?

Your participation will help examine 1) the status of your school's social mission activities; 2) current strengths and areas for improvement in these activities; and 3) your school's proficiency in social mission in comparison to similar schools. In addition to the confidential summary report, the data from the self-assessment will enable the research team to further develop performance measures in the field of social mission.

Who is sponsoring this study?

This project is funded by a research grant from the Robert Wood Johnson Foundation.

How should school-directed questions be answered?

Please answer school-directed questions in relation to the School of Medicine. For example, if your individual school is situated in a larger university or health science center, focus on the individual degree granting school/college for your MD or DO students where possible.

What if I do not understand a term?

Text boxes throughout the document define phrases used in the study, and there is a glossary at the end included for reference. If there are other questions or terms you do not understand, please email Julie Orban (juliela@gwu.edu) for further clarification.

© 2022 Batra S et al. *JAMA Network Open*.

YOUR SCHOOL'S EDUCATIONAL PROGRAM

A. Curricular courses and programs

A1. What is the primary degree that you offer at your medical school?

1 Doctor of Medicine (MD)
2 Doctor of Osteopathic Medicine (DO)

A2. Does your school have required inter-professional education for all students?

1 Yes
2 No
9 *Don't know*

A2a. **IF YES:** In which settings do students receive inter-professional education experiences?

1 In clinical settings only
2 In classroom/simulation settings only
3 In both clinical and classroom/simulation settings
9 *Don't know*

A3. Does your school offer clinical rotations or courses where your students interact with patients from underserved communities?

1 Yes
2 No
9 *Don't know*

IF YES: Please answer A3a-A3c:

A3a. Are these clinical rotations or courses required of students?

1 Yes, required of all our students
2 Yes, required of certain students
3 No

A3b. **IF EXPERIENCES ARE NOT REQUIRED:** About how many of your students participate in these clinical rotations or courses over the course of their education

1 All or nearly all (91% or more)
2 Most (51-90%)
3 Some (10-50%)
4 Just a few (less than 10%)
9 *Don't know*

A3c. Are any of these clinical rotations or courses longitudinal i.e. clinical engagements over a prolonged period of time (at least four weeks of student time in the period of a year)?

1 Yes
2 No
9 *Don't know*

© 2022 Batra S et al. *JAMA Network Open*.

> **Definition: Social Determinants of Health** are the complex, integrated, and overlapping social structures and economic systems that are responsible for most health inequities. These social structures and economic systems include the social environment, physical environment, health services, and structural and societal factors.[1]

A4. Does your school explicitly teach students about social determinants of health in your curriculum?

1 Yes, in required courses
2 Yes, in elective courses
3 No

A4a. **IF YES:** Is this content integrated across multiple years of study?

1 Yes, across all years of study
2 Yes, across multiple years of study
3 No

> **Definition: Health disparities** are preventable differences in the burden of disease, injury, violence, or opportunities to achieve optimal health that are experienced by socially disadvantaged populations. Populations can be defined by factors such as race or ethnicity, gender, education or income, disability, geographic location (e.g., rural or urban), or sexual orientation. Health disparities are inequitable and are directly related to the historical and current unequal distribution of social, political, economic, and environmental resources.[2]

A5. Does your school explicitly teach students about health disparities in your curriculum?

1 Yes, in required courses
2 Yes, in elective courses
3 No

A5a. **IF YES:** Is this content integrated across multiple years of study?

1 Yes, across all years of study
2 Yes, across multiple years of study
3 No

[1] NCHHSTP social determinants of health. Centers for Disease Control and Prevention website. https://www.cdc.gov/nchhstp/socialdeterminants/definitions.html. Updated Marched 21, 2014. Accessed November 9, 2017.
[2] Disparities. Centers for Disease and Control and Prevention website. https://www.cdc.gov/healthyyouth/disparities/. Updated September 1, 2015. Accessed November 30, 2017.

© 2022 Batra S et al. *JAMA Network Open*.

Definition: LGBTQ is an acronym for lesbian, gay, bisexual, transgender, and queer.[3]

We understand that terminology and acronyms continue to evolve. Please use whatever terminology your school finds most appropriate.

A6. Does your school have specific curricular content focused on LGBTQ health?

1 Yes, in required courses
2 Yes, in elective courses
3 No

A6a. **IF YES:** Is this content integrated across multiple years of study?

1 Yes, across all years of study
2 Yes, across multiple years of study
3 No

[3] GLAAD media reference guide - Lesbian / gay / bisexual glossary of terms. GLAAD website. https://www.glaad.org/reference/lgbtq. Accessed November 29, 2017.

© 2022 Batra S et al. *JAMA Network Open*.

B. Extracurricular and service learning opportunities

B1. Does your school have a service-learning or volunteer hours requirement for graduation?

1 Yes, required of all students
2 Yes, required of some students
3 No

B1a. **IF EXPERIENCES ARE NOT REQUIRED:** About how many of your students participate in service-learning or volunteer opportunities during the course of their education?

1 All or nearly all (approximately 91% or more)
2 Most (51-90%)
3 Some (11-50%)
4 Just a few (10% or less)
9 *Don't know*

B1b. **IF EXPERIENCES ARE REQUIRED:** What is the total time commitment required?

1 No specific time commitment
2 1-10 hours
3 11-30 hours
4 31-50 hours
5 > 50 hours

B2. Other than student-run health clinics, does your school offer its students extracurricular activities directly related to reduction of health disparities or addressing social determinants of health?

1 Yes
2 No
9 *Don't know*

B2a. **IF YES:** About how many of your students participate in these experiences during the course of their education?

1 All or nearly all (91% or more)
2 Most (51-90%)
3 Some (10-50%)
4 Just a few (less than 10%)
5 None
9 *Don't know*

© 2022 Batra S et al. *JAMA Network Open*.

C. Targeted education programs

C1. Does your institution or school offer a certification or degree in Public Health?

1 Yes
2 No
9 *Don't know*

C1a. **IF YES:** Approximately what percentage of your medical students over the last four graduating classes have completed the Public Health certification or degree option?

1 All or nearly all (91% or more)
2 Most (51-90%)
3 Some (10-50%)
4 Just a few (less than 10%)
5 None
9 *Don't know*

C2. Does your institution or school offer specific education programs that train students to enter the following health careers?

**Do you offer a program that trains students to become . . .**	Yes	No	*Don't know*
a. Community Health Workers	1	2	*9*
b. Medical Assistants	1	2	*9*

© 2022 Batra S et al. *JAMA Network Open*.

D. Global health

D1. Does your school have a formal partnership with an institution in a low or middle income country?

1 Yes
2 No
9 *Don't know*

D1a. **IF YES:** Does your school facilitate students from low or middle income countries to train or study at your institution?

1 Yes
2 No
9 *Don't know*

D2. Does your school offer global health rotations in low or middle income countries?

1 Yes
2 No
9 *Don't know*

IF YES: Please answer D2a-D2b:

D2a. About how many of your students participate in these experiences?

1 All or nearly all (91% or more)
2 Most (51-90%)
3 Some (10-50%)
4 Just a few (less than 10%)
9 *Don't know*

D2b. What is the average duration of the experience?

1 < 2 weeks
2 2-4 weeks
3 > 4 weeks

© 2022 Batra S et al. *JAMA Network Open*.

E. Statements related to your school's mission

Definition: Community of commitment is the term we are using to indicate a medically or socially underserved community – this could be an underserved geographic area (local or regional), demographic group, or category of patient that your school has explicitly targeted as a focus for your work.

E1. Does your school have a written mission statement? *Please circle a number.*

1 Yes
2 No

E1a. **IF YES:** Does your school's mission statement specifically mention any of the following terms (*select all that apply*):

- ❑ Social determinants of health
- ❑ Underserved/underrepresented/disadvantaged populations
- ❑ Health equity or health disparities

E1b. **IF YES:** Does your school's mission statement identify a specific "community of commitment" as defined above? If you're not sure, please send us your mission statement or give us the Web address (URL) where we can locate it and we will attempt to make a determination.

1 Yes
2 No
9 *Don't know*

URL:

E1c. **IF YES:** Please specify your school's community or communities of commitment:

E2. Does your school have a current strategic plan?

1 Yes
2 No

E2a. **IF YES:** Does your school's strategic plan specifically mention any of the following terms (*select all that apply*):

- ❑ Social determinants of health
- ❑ Underserved/underrepresented/disadvantaged populations
- ❑ Health equity or health disparities

E2b. **IF YES:** Does your strategic plan identify a specific "community of commitment" as defined above? If you're not sure, please send us your strategic plan and we will attempt to make a determination.

1 Yes
2 No
9 *Don't know*

E2c. **IF YES:** Please specify your school's community or communities of commitment:

© 2022 Batra S et al. *JAMA Network Open*.

F. Concordance of curriculum with community needs

Definition: A Community health needs assessment (sometimes called a CHNA), refers to a state, tribal, local, or territorial health assessment that identifies key health needs and issues through systematic, comprehensive data collection and analysis.[4]

F1. Has your school, or a hospital with which it is closely affiliated, conducted a formal or informal Community Health Needs Assessment in the past five years?

1 Yes, formal
2 Yes, informal
3 Neither
9 *Don't know*

F1a. **IF YES:** To what extent has the design of your school's curriculum been explicitly informed by the results of the Community Health Needs Assessment?

1 Substantially
2 Moderately
3 Slightly
4 Not at all
9 *Don't know*

[4] Community health assessments & health improvement plans. Centers for Disease Control and Prevention website. https://www.cdc.gov/stltpublichealth/cha/plan.html. Updated November 9, 2015. Accessed August 29, 2016.

© 2022 Batra S et al. *JAMA Network Open*.

G. Community collaborations

G1. Do your students participate in clinics, offices, or programs that are located outside of your main campus and based in the local community or in your communities of commitment?

1 Yes
2 No
9 *Don't know*
10 *Not applicable*

G1a. **IF YES:** Does your school provide any compensation for time devoted by faculty or staff to the activities in these off-campus, community locations?

1 Yes
2 No
9 *Don't know*

G1b. Is there a formal or informal channel for feedback ***from the community to the school*** about the value and effectiveness of these programs?

1 Yes
2 No
9 *Don't know*

G2. Does your school have formalized or otherwise well-developed collaborations with any of the following? *Please circle a number for each type.*

**Type of collaboration**	Yes	No	*Don't know/ Not applicable*
a. Collaborations with legal professionals	1	2	*9*
b. Collaborations with churches, temples or other faith-based organizations	1	2	*9*
c. Collaborations, practice arrangements, or clinical teaching arrangements with a local Federally Qualified Health Center (FQHC)	1	2	*9*
d. Collaborations with local K-12 schools	1	2	*9*
e. Collaborations with local community colleges	1	2	*9*
f. Collaboration with your local government's health department	1	2	*9*
g. Collaboration with philanthropic organizations	1	2	*9*

© 2022 Batra S et al. *JAMA Network Open*.

H. Student diversity

Definition: Holistic review is a university admissions strategy that assesses an applicant's unique experiences alongside traditional measures of academic achievement such as grades and test scores. It is designed to help universities consider a broad range of factors reflecting the applicant's academic readiness, contribution to the incoming class, and potential for success both in school and later as a professional.[5] Holistic Review has been described by the Supreme Court as a highly individualized review of each applicant's file, giving serious consideration to all the ways an applicant might contribute to a diverse educational environment.[6]

H1. Does your program's admission policy include principles of holistic review of applicants?

1 Yes, as a matter of written policy
2 Yes, as a matter of informal practice
3 No
9 *Don't know*

H2. What is the total number of students currently enrolled in your degree program?

Total number of students: ______

[5] Urban Universities for HEALTH. Holistic admissions in the health professions: findings from a national survey. http://urbanuniversitiesforhealth.org/media/documents/holisticadmissionsinthehealthprofessions.pdf. Published September 2014. Accessed September 1, 2016.

[6] Supreme Court of the United States. Grutter v. Bollinger. https://www.supremecourt.gov/opinions/boundvolumes/539bv.pdf. Published 2005. Accessed August 29, 2016.

© 2022 Batra S et al. *JAMA Network Open*.

H3. Please fill in a percentage breakdown of the racial and ethnic composition of your current student body in the table below. Please include all full-time students currently enrolled in your primary degree program. (*Note: This should be self-reported race and ethnicity data that is on file with your school. Please enter zeros in blanks.)*

**Percent of your *full-time students* who identify as:***	Percent
a. Hispanic or Latino	____%
b. American Indian or Alaskan Native (not Hispanic)	____%
c. Asian (not Hispanic)	____%
d. Black or African American (not Hispanic)	____%
e. Native Hawaiian or Other Pacific Islander	____%
f. White (not Hispanic)	____%
g. Two or more races (not Hispanic)	____%
h. Race/ethnicity not reported/not known	____%
i. International student (Nonresident alien)	____%
Total (should total to 100%)	_____%

*The categories in this table are based on those that schools report to the U.S. Dept. of Education

H4. What is the average graduation rate for students enrolled in your primary degree program over the past 5 cohorts? *Note: If you do not have data over the past 5 cohorts, use whatever time frame you have available to calculate the average rate. You will be asked to specify the time frame below.*

Overall graduation rate: ______%

This rate is calculated or estimated over the past ______ years.

This rate represents the percentage of students who graduated within _____ years.

© 2022 Batra S et al. *JAMA Network Open*.

H5. What is the average graduation rate for students by race/ethnicity enrolled in your primary degree program over the past 5 cohorts? *Note: These rates should be calculated over the same time period as the question above. These numbers should NOT add up to 100%. For example: If you are using a time period of 5 years, and your last 5 classes included 10 self-identified white students, 9 of whom graduated, your answer should be 90%.*

	American Indian or Alaskan Native	Asian/ Pacific Islander	Black	Hispanic	White	Two or more races	*Race Not Reported*
Graduation rate:	_____%	_____%	_____%	_____%	_____%	_____%	_____%

This rate is calculated or estimated over the past ______ years.

This rate represents the percentage of students who graduated within _____ years.

H6. Approximately what percentage of your students in your primary degree program are from the community of commitment referred to in section E?

Percent of students: _____%

❑ *Information not available*

H7. What percentage of students in your primary degree program are from families where neither parent finished college?

Percent of students who were first-generation college students: _____%

❑ *Information not available*

H7a. Considering only first-generation college students in your primary degree program, what is their average graduation rate? *Note: This rate should be calculated over the same time period as the questions above.*

Graduation rate for first-generation college students: _____%

❑ *Information not available*

H8. Please consider all the scholarship and grant dollars awarded by your school in the last academic year. What percentage of the scholarship and grant dollars were awarded based on financial need? *(Do not count student loans or student wages.)*

Percent of scholarship and grant dollars that are need-based: ______%

❑ *Information not available*

© 2022 Batra S et al. *JAMA Network Open*.

Since definitions and abbreviations are evolving, we may have omitted some letters from the LGBTQ abbreviation. Please apply your schools practice to the next section as you feel appropriate.

H9. Does your school collect information from each student on his or her self-reported sexual orientation and/or preferred gender identity?

1 Yes
2 No

H9a. **IF YES:** What percentage of students in your primary degree program self-identify as LGBTQ?

Percent of students identifying as LGBTQ: _____%

❑ *Information not available*

© 2022 Batra S et al. *JAMA Network Open*.

J. Faculty diversity

The following questions ask about the composition of your faculty. Please include all **full-time** faculty members in your school, regardless of rank or tenure status. Faculty members on joint appointments with other schools should be included if the appointment in your school is considered to be their primary appointment. Adjunct faculty should be included if their work commitment to the school is considered to be full-time.

J1. What is the total number of full-time faculty at your school?

Total number of full-time faculty: ______

J2. Please fill in a percentage breakdown of the racial and ethnic composition of your school's *full-time faculty* in the table below. *(This should be self-reported race and ethnicity data that is on file with your school. Please enter zeros in blanks.)*

❑ *Information not available*

**Percent of your school's *full-time faculty* who identify as:**	Percent
a. Hispanic or Latino	____%
b. American Indian or Alaskan Native (not Hispanic)	____%
c. Asian (not Hispanic)	____%
d. Black or African American (not Hispanic)	____%
e. Native Hawaiian or Other Pacific Islander	____%
f. White (not Hispanic)	____%
g. Two or more races (not Hispanic)	____%
h. Race/ethnicity not reported/not known	____%
i. International employee (Nonresident alien)	____%
Total (should total to 100%)	_____%

*The categories in this table are based on those that schools report to the U.S. Dept. of Education

© 2022 Batra S et al. *JAMA Network Open*.

J3. What percentage of your *full-time faculty* members are women?

Percent of women among all full-time faculty: _____%

J4. Does your *full-time faculty* team include one or more members who openly identify themselves as members of the LGBTQ community?

1 None
2 1-2
3 3-4
4 5-9
5 10 or more
9 *Don't know*

The next questions are about the people in **academic leadership** positions in your school. Please include members of the faculty who hold decanal positions (e.g. dean, assistant/associate dean), provost, department chair, division head, or the equivalent positions at your school. Do not include non-faculty administrators.

J5. What is the total number of academic leadership members at your school?

Total number of faculty in academic leadership: ______

© 2022 Batra S et al. *JAMA Network Open*.

J6. Please fill in a percentage breakdown of the racial and ethnic composition of your school's academic leadership in the table below. *(This should be self-reported race and ethnicity data that is on file with your school. Please enter zeros for blanks.)*

❑ *Information not available*

**Percent of your school's *academic leadership* who identify as:**	Percent
a. Hispanic or Latino	____%
b. American Indian or Alaskan Native (not Hispanic)	____%
c. Asian (not Hispanic)	____%
d. Black or African American (not Hispanic)	____%
e. Native Hawaiian or Other Pacific Islander	____%
f. White (not Hispanic)	____%
g. Two or more races (not Hispanic)	____%
h. Race/ethnicity not reported/not known	____%
i. International employee (Nonresident alien)	____%
Total (should total to 100%)	_____%

*The categories in this table are based on those that schools report to the U.S. Dept. of Education

J7. What percentage of your *academic leadership* members are women?

Percent of women among academic leadership: _____%

J8. Does your *academic leadership* team include any member who openly identify themselves as members of the LGBTQ community?

1 Yes
2 No
9 *Don't know*

© 2022 Batra S et al. *JAMA Network Open*.

K. Pipeline programs

K1. Is your school sponsoring, running or assisting with any pipeline programs targeted to K-12 students and aimed at encouraging them to train for careers in the health professions?

1 Yes
2 No
9 *Don't know*

IF YES: Please answer K1a-K1c:

K1a. Other than one-time contacts with students, what is the number of K-12 students who participate in your pipeline programs each year?

Number of K-12 students impacted annually: ____

K1b. Of the answer you gave in K1a, approximately what percentage of these students are from underrepresented racial/ethnic minority groups?

Percent of participating K-12 students who are minorities: _____*%*

K1c. Of the answer you gave in K1a, approximately what percentage of these students are in the free or reduced lunch program?

Percent of participating K-12 students who are on free or reduced lunch: _____*%*

K2. Is your school sponsoring, running or assisting with any pipeline programs targeted to undergraduate college students and aimed at encouraging them to train for careers in the health professions?

1 Yes
2 No
9 *Don't know*

IF YES: Please answer K2a-K2d:

K2a. Other than one-time contacts with students, what is the number of undergraduate students who participate in your pipeline programs each year?

Number of undergraduate students impacted annually: ____

K2b. Approximately what percentage of the participating students are from underrepresented racial/ethnic minority groups?

Percent of participating undergraduate students who are minorities: _____*%*

K2c. Approximately what percentage of the participating students are the first in their family to go to college?

Percent of participating students who are first-generation college students: _____*%*

K2d. Approximately what percentage of the participating students ultimately enter education for one of the health professions? *(Note: Do not include students enrolled in pre-matriculation who have already been admitted to a health professions school.)*

Percent of participating students who enter health profession education: _____*%*

© 2022 Batra S et al. *JAMA Network Open*.

L. Training for a culture of inclusion

L1. How many of your current full-time faculty have taken any of the following types of training?

Training in . . .	All or nearly all (91% or more)	Most (51-90%)	Some (10-50%)	Just a few (less than 10%)	*Not Offered*	*Don't Know*
a. Unconscious/implicit bias awareness	1	2	3	4	*5*	*9*
b. Cultural competency or cultural humility	1	2	3	4	*5*	*9*
c. Advocacy on issues related to health	1	2	3	4	*5*	*9*
d. Social determinants of health	1	2	3	4	*5*	*9*

L2. How many of your current students have taken any of the following types of training?

Training in . . .	All or nearly all (91% or more)	Most (51-90%)	Some (10-50%)	Just a few (less than 10%)	*Not Offered*	*Don't Know*
a. Unconscious/implicit bias awareness	1	2	3	4	*5*	*9*
b. Cultural competency or cultural humility	1	2	3	4	*5*	*9*
c. Advocacy on issues related to health	1	2	3	4	*5*	*9*
d. Social determinants of health	1	2	3	4	*5*	*9*

© 2022 Batra S et al. *JAMA Network Open*.

M. Student-run health clinics

Definition: Student-run health clinics are community clinics where students (with faculty supervision) provide health care services at no cost or low cost to underserved populations.

M1. In the past three years, have your students worked in any student-run health clinics?

1 Yes
2 No
9 *Don't know*

M1a. **IF YES:** About how many of your students volunteer at your student-run health clinics?

1 All or nearly all (91% or more)
2 Most (51-90%)
3 Some (10-50%)
4 Just a few (less than 10%)
5 None

M1b. **IF YES:** About how many of your faculty or staff members actively give time to your student-run health clinics?

1 All or nearly all (91% or more)
2 Most (51-90%)
3 Some (10-50%)
4 Just a few (less than 10%)
5 None

M1c. **IF YES:** Does your school provide compensation for any of the time that faculty or staff devote to your student-run health clinics?

1 Yes
2 No
9 *Don't know*

© 2022 Batra S et al. *JAMA Network Open*.

N. Student activism

N1. Other than time given to student-run health clinics, about how many of your *students* are actively involved in community-based programs or organizations that address health disparities or social determinants of health?

1 All or nearly all (91% or more)
2 Most (51-90%)
3 Some (10-50%)
4 Just a few (less than 10%)
5 None
9 *Don't know*

N2. Does your school provide any regular financial support for community-oriented student activities outside of the curriculum (not including student-run health clinics)?

1 Yes
2 No
9 *Don't know*

N3. Consider your students who are from under-represented groups in the health professions. About how many of these students are active in minority professional associations and societies?

1 All or nearly all (91% or more)
2 Most (51-90%)
3 Some (10-50%)
4 Just a few (less than 10%)
5 None
9 *Don't know*

© 2022 Batra S et al. *JAMA Network Open*.

P. Faculty activism

P1. Other than time given to student-run health clinics, about how many of your *faculty* are actively involved in community-based programs or organizations that address health or issues related to health?

1 All or nearly all (91% or more)
2 Most (51-90%)
3 Some (10-50%)
4 Just a few (less than 10%)
5 None
9 *Don't know*

P2. About how many of your faculty members are actively involved in advocating for change in issues related to health or its social determinants? *(Include regional, state, national or international advocacy organizations.)*

1 All or nearly all (91% or more)
2 Most (51-90%)
3 Some (10-50%)
4 Just a few (less than 10%)
5 None
9 *Don't know*

P3. Does your school recognize or reward faculty participation in the activities in P1 or P2?

1 Yes
2 No
9 *Don't know*

P3a. **IF YES:** How does your school recognize or reward faculty?

1 Promotion criteria explicitly recognize these contributions
2 Awards for community activities or advocacy
3 Compensation/pay raise
4 Other _____

© 2022 Batra S et al. *JAMA Network Open*.

Q. Emphasis on primary care

Q1. How would you rate your school's amount of encouragement or discouragement for students to enter primary care or general community-based practice?

1 A great deal of encouragement
2 Some encouragement
3 A little encouragement
4 Neither encourage or discourage
5 A little discouragement
6 Some discouragement
7 A great deal of discouragement

Q2. Approximately what percentage of your graduates practice in community health centers, including Federally Qualified Health Centers (FQHCs)?

Percent of graduates: _____%

❑ *Information not available*

Q3. Over the past four years, approximately what percentage of your graduates typically enter residency in family practice?

Percent of graduates who enter residency in family practice: _____%

❑ *Information not available*

Q4. Approximately what percentage of your graduates typically enter primary care practice <u>following</u> residency (family medicine, general internal medicine, general pediatrics, med-peds, or general ob-gyn)?

Percent of graduates who enter primary care: _____%

❑ *Information not available*

© 2022 Batra S et al. *JAMA Network Open*.

R. Community needs and social mission in your school's research

Definitions: Community based participatory research is a collaborative approach to research that equitably involves all partners in the research process and recognizes the unique strengths that each brings. CBPR begins with a research topic of importance to the community and has the aim of combining knowledge with action and achieving social change to improve health outcomes and eliminate health disparities.[7]

Community engaged research is a process of inclusive participation that supports mutual respect of values, strategies, and actions for authentic partnership of people *affiliated with* or *self-identified* by geographic proximity, special interest, or similar situations to address issues affecting the well-being of the *community of focus*.[8]

Health equity research is research that examines strategies, programs or circumstances that tend to reduce health disparities and achieve optimal health for all.[9]

Health disparity research includes basic, clinical and social sciences studies that focus on identifying, understanding, preventing, diagnosing, and treating health conditions such as diseases, disorders, and other conditions that are unique to, more serious, or more prevalent in subpopulations in socioeconomically disadvantaged (i.e., low education level, live in poverty) and medically underserved, rural, and urban communities.[10]

Health promotion research is research that promotes the process of enabling people to increase control over, and to improve, their health. It moves beyond a focus on individual behavior towards a wide range of social and environmental interventions.[11]

Disease prevention research is research designed to yield results directly applicable to identifying and assessing risk, and to developing interventions for preventing or ameliorating the

[7] Faridi Z, Grunbaum JA, Gray BS, Franks A, Simoes E. Prev Chronic Dis. 2007;4(3):2. https://www.cdc.gov/pcd/issues/2007/jul/pdf/06_0182.pdf. Published July 2007. Accessed August 29, 2016.
[8] Ahmed, SM, Palermo, AG. Community Engagement in Research: Frameworks for Education and Peer Review. Am J Public Health. 2010;100(8): 1380-7. https://ajph.aphapublications.org/doi/full/10.2105/AJPH.2009.178137. Accessed January 14, 2019.
[9] Health equity. Centers for Disease Control and Prevention website. https://www.cdc.gov/chronicdisease/healthequity/. Updated February 10, 2015. Accessed August 29, 2016.
[10] Health disparities definition. National Institutes of Health website. https://www.drugabuse.gov/about-nida/organization/health-disparities/about-nida-health-disparities/nih-health-disparities-definition. Accessed August 29, 2016.
[11] Health promotion. World Health Organization website. http://www.who.int/topics/health_promotion/en/. Accessed August 29, 2016.

© 2022 Batra S et al. *JAMA Network Open*.

occurrence of a disease or the progression of detectable but asymptomatic disease.[12]

R1. For each of the following types of research, please tell us the number of research projects that your school have been involved in during the past three years. If appropriate, you may count a given project under more than one applicable type of research. *Please circle a response for each type of research.*

**How many of these projects in the last 3 years?**	None	One	2 or 3	4 to 9	10 or more	*Don't know*
a. Community engaged research (including community based participatory research)	0	1	2-3	4-9	10+	*9*
b. Health equity or health disparity research	0	1	2-3	4-9	10+	*9*
c. Health promotion or disease prevention research	0	1	2-3	4-9	10+	*9*
d. Research into social determinants of health	0	1	2-3	4-9	10+	*9*
e. Health or research needs assessment in your school's local community or communities of commitment	0	1	2-3	4-9	10+	*9*

R2. Approximately what percentage of your school's total research portfolio is focused on research in all of the above categories combined (community engaged research, health equity/disparity research, health promotion/disease prevention research, social determinants of health, community health needs assessment)

1 0-10%
2 11-25%
3 26-50%
4 > 50%

R3. To what extent has the development of your school's research program been explicitly informed by the results of your school or hospital's Community Health Needs Assessment or other ongoing mechanisms designed to monitor and respond to community health priorities?

1 Substantially
2 Moderately
3 Slightly
9 Don't know
10 Our school doesn't have a Community Health Needs Assessment

[12] Prevention research at NIH. National Institutes of Health website. https://prevention.nih.gov/prevention-research. Updated April 8, 2016. Accessed August 29, 2016.

© 2022 Batra S et al. *JAMA Network Open*.

CLOSING COMMENTS

THANK YOU for sharing so much information about your school's programs and efforts to advance the social mission in health professions education. Your responses are invaluable to our research and our efforts to advance these areas in the education of our nation's health professionals.

We would like to hear more from you about what your school is doing in the area of social mission. Please take a moment to answer the following question:

S. Your school's social mission activities

S1. What projects or ideas is your school working on in the next 3 years that is related to social mission?

Thanks again for completing the assessment.

© 2022 Batra S et al. *JAMA Network Open*.

GLOSSARY

Community based participatory research is a collaborative approach to research that equitably involves all partners in the research process and recognizes the unique strengths that each brings. CBPR begins with a research topic of importance to the community and has the aim of combining knowledge with action and achieving social change to improve health outcomes and eliminate health disparities.

Community engaged research is a process of inclusive participation that supports mutual respect of values, strategies, and actions for authentic partnership of people *affiliated with* or *self-identified by* geographic proximity, special interest, or similar situations to address issues affecting the well-being of the *community of focus*.

Community health needs assessment (sometimes called a CHNA), refers to a state, tribal, local, or territorial health assessment that identifies key health needs and issues through systematic, comprehensive data collection and analysis.

Community of commitment is the term we are using to indicate a medically or socially underserved community – a health disparity community – that could be a geographic area (local or regional), demographic group, or category of patient that your school has explicitly targeted as a focus for your work.

Disease prevention research is research designed to yield results directly applicable to identifying and assessing risk, and to developing interventions for preventing or ameliorating the occurrence of a disease or the progression of detectable but asymptomatic disease.

Health equity research is research that aims to eliminate health disparities and achieve optimal health for all.

Health disparities are preventable differences in the burden of disease, injury, violence, or opportunities to achieve optimal health that are experienced by socially disadvantaged populations. Populations can be defined by factors such as race or ethnicity, gender, education or income, disability, geographic location (e.g., rural or urban), or sexual orientation. Health disparities are inequitable and are directly related to the historical and current unequal distribution of social, political, economic, and environmental resources.

Health disparity research includes basic, clinical and social sciences studies that focus on identifying, understanding, preventing, diagnosing, and treating health conditions such as diseases, disorders, and other conditions that are unique to, more serious, or more prevalent in subpopulations in socioeconomically disadvantaged (i.e., low education level, live in poverty) and medically underserved, rural, and urban communities.

Health promotion research is research that promotes the process of enabling people to increase control over, and to improve, their health. It moves beyond a focus on individual behavior towards a wide range of social and environmental interventions.

Holistic review is a university admissions strategy that assesses an applicant's unique experiences alongside traditional measures of academic achievement such as grades and test scores. It is designed to

© 2022 Batra S et al. *JAMA Network Open*.

help universities consider a broad range of factors reflecting the applicant's academic readiness, contribution to the incoming class, and potential for success both in school and later as a professional. Holistic Review has been described by the Supreme Court as a highly individualized review of each applicant's file, giving serious consideration to all the ways an applicant might contribute to a diverse educational environment.

LGBTQ is an acronym for lesbian, gay, bisexual, transgender, and queer.

Social determinants of health are the complex, integrated, and overlapping social structures and economic systems that are responsible for most health inequities. These social structures and economic systems include the social environment, physical environment, health services, and structural and societal factors.

Student-run health clinics are community clinics where students (with faculty supervision) provide health care services at no cost or low-cost to underserved populations.

© 2022 Batra S et al. *JAMA Network Open*.

eFigure 2. Frequency Histogram of Participating Self-Assessment Dental, Medical, and Nursing Schools' Overall Social Mission Score, 2019[a]

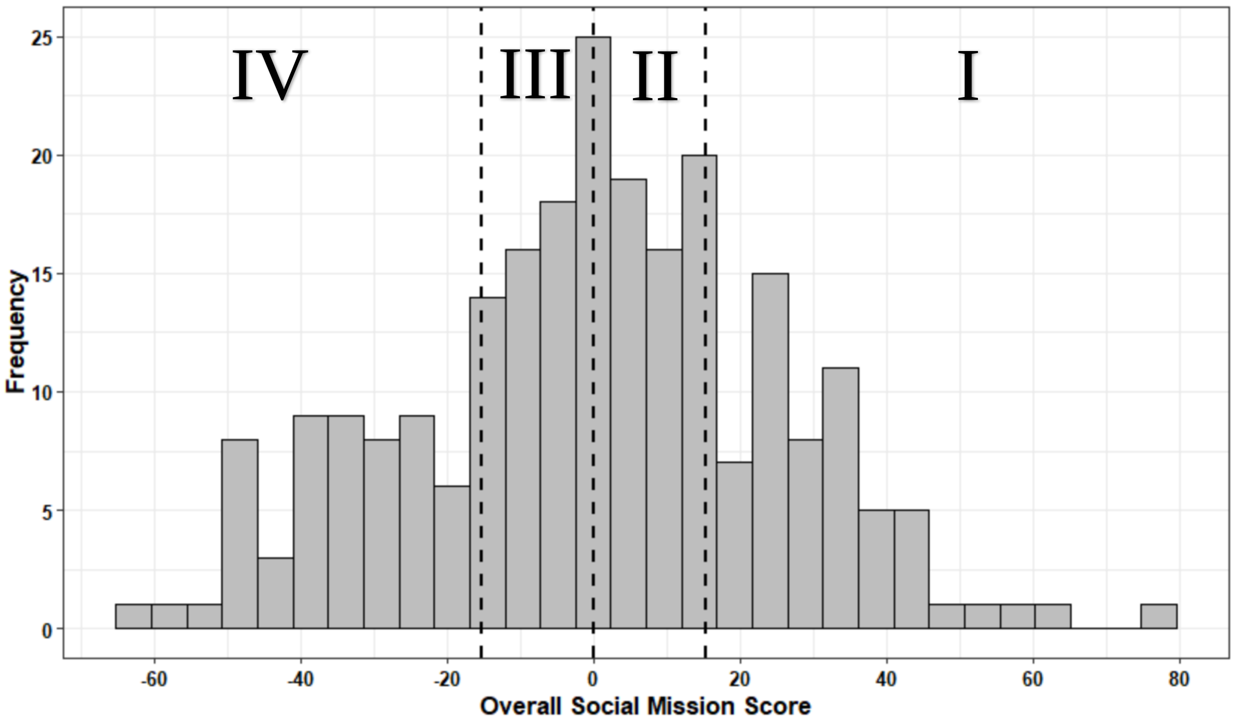

[a]The dotted lines separate the top (I), second (II), third (III) and bottom (IV) quartiles of schools in the Social Mission Metrics Self-Assessment Survey, 2019.

© 2022 Batra S et al. *JAMA Network Open*.

eFigure 3. Standardized Area Scores by Health Professions Discipline From the Social Mission Metrics National Self-Assessment for Areas 13 to 18, 2019[a,b]

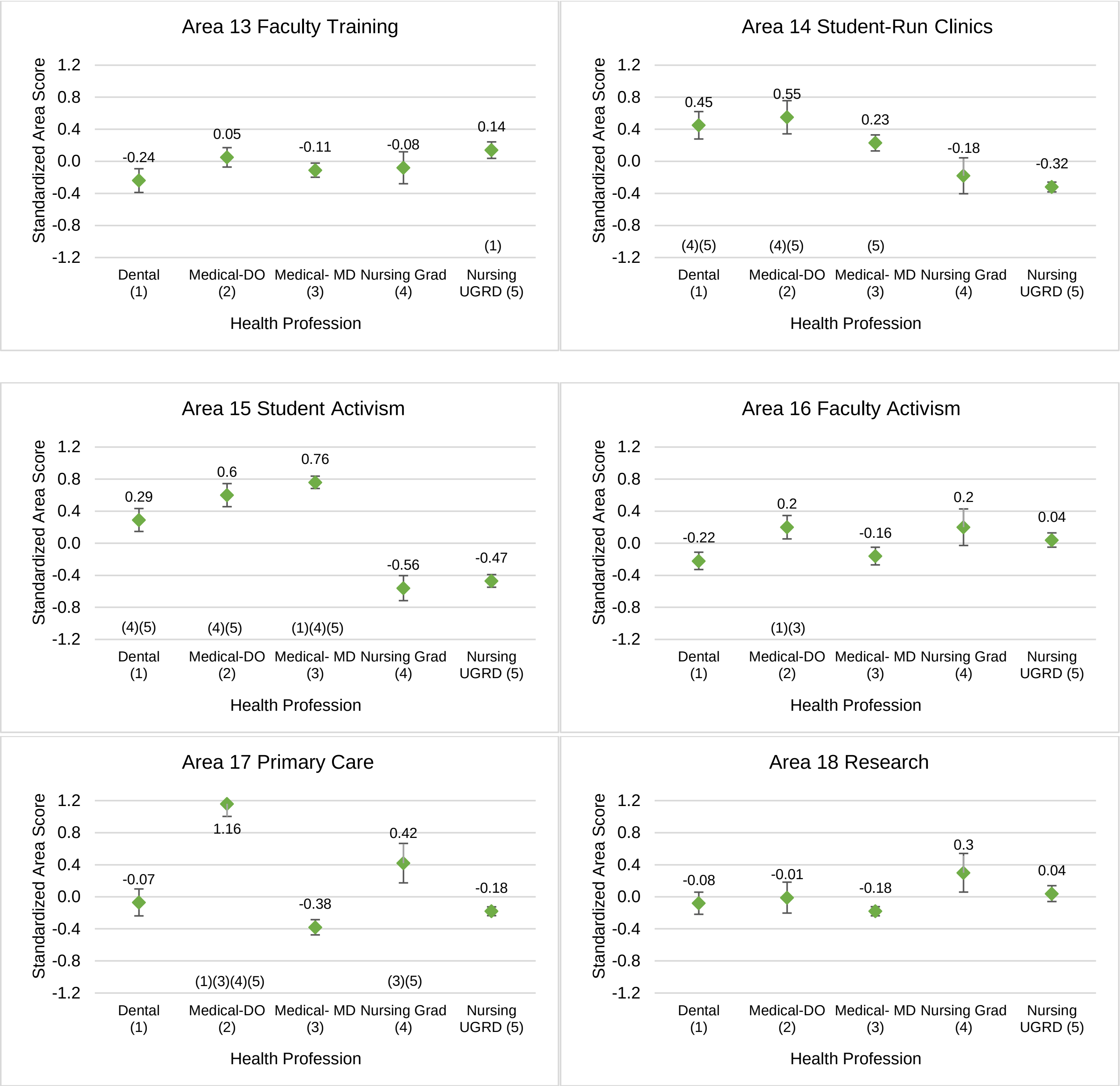

[a](1)(2)(3)(4)(5) number indicate the five health profession groups. Numbers above the x axis indicate the groups whose means are significantly LOWER than this group's mean, based on pairwise t-tests with a finite population correction and Fisher's LSD correction. For example, in Area 15 (Student Activism), group 2 (DO medical schools) and group 3 (MD medical schools) scores significantly higher than each of the three other groups.

[b]There were 26 dental schools, 25 DO Medical schools, 58 MD Medical schools, 31 graduate nursing schools, and 102 undergraduate nursing schools included in the analysis.

© 2022 Batra S et al. *JAMA Network Open*.

eFigure 4. Standardized Area Scores by Carnegie Classification From the Social Mission Metrics National Self-Assessment for Areas 1 to 18, 2019[a,b,c]

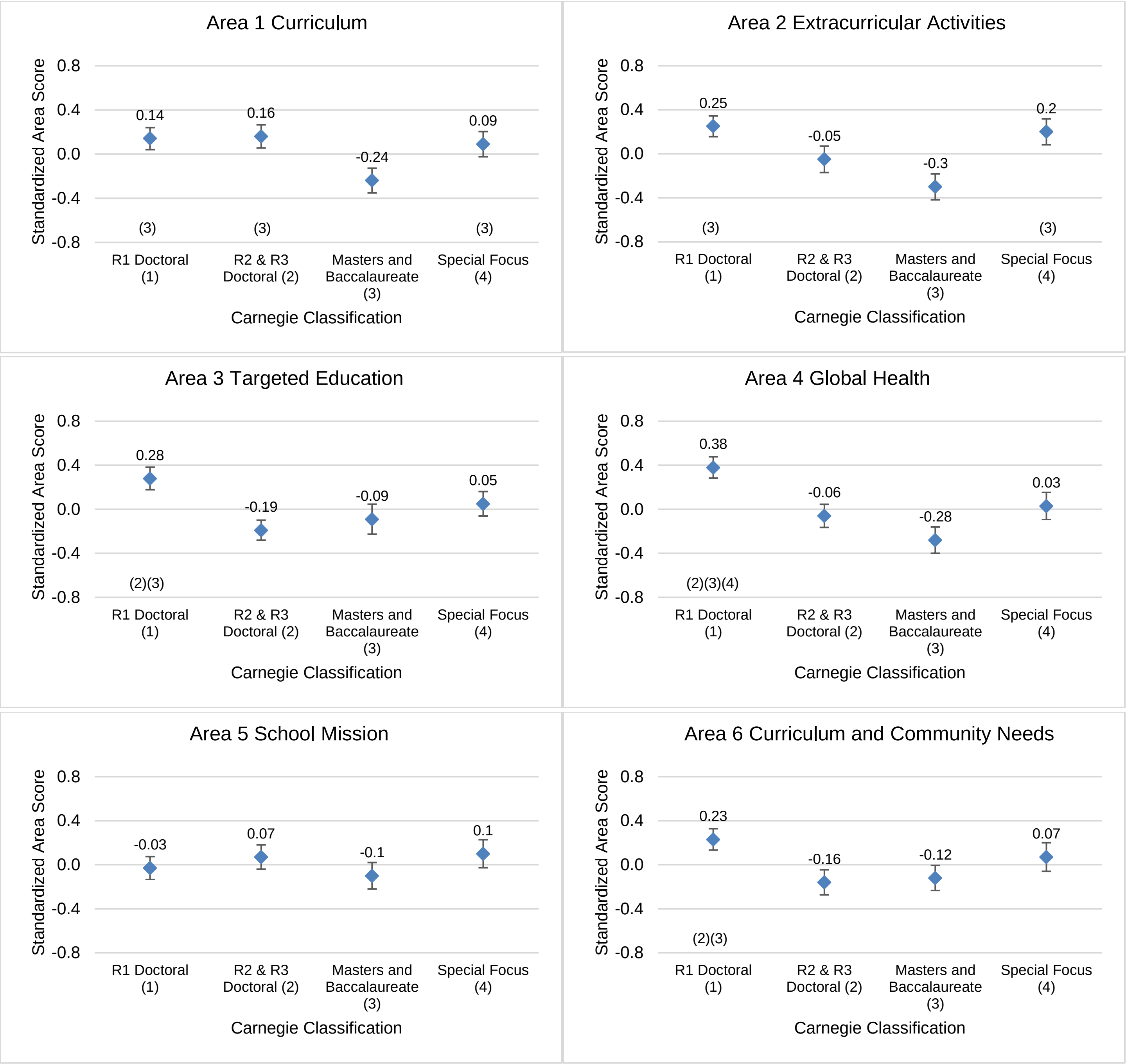

© 2022 Batra S et al. *JAMA Network Open*.

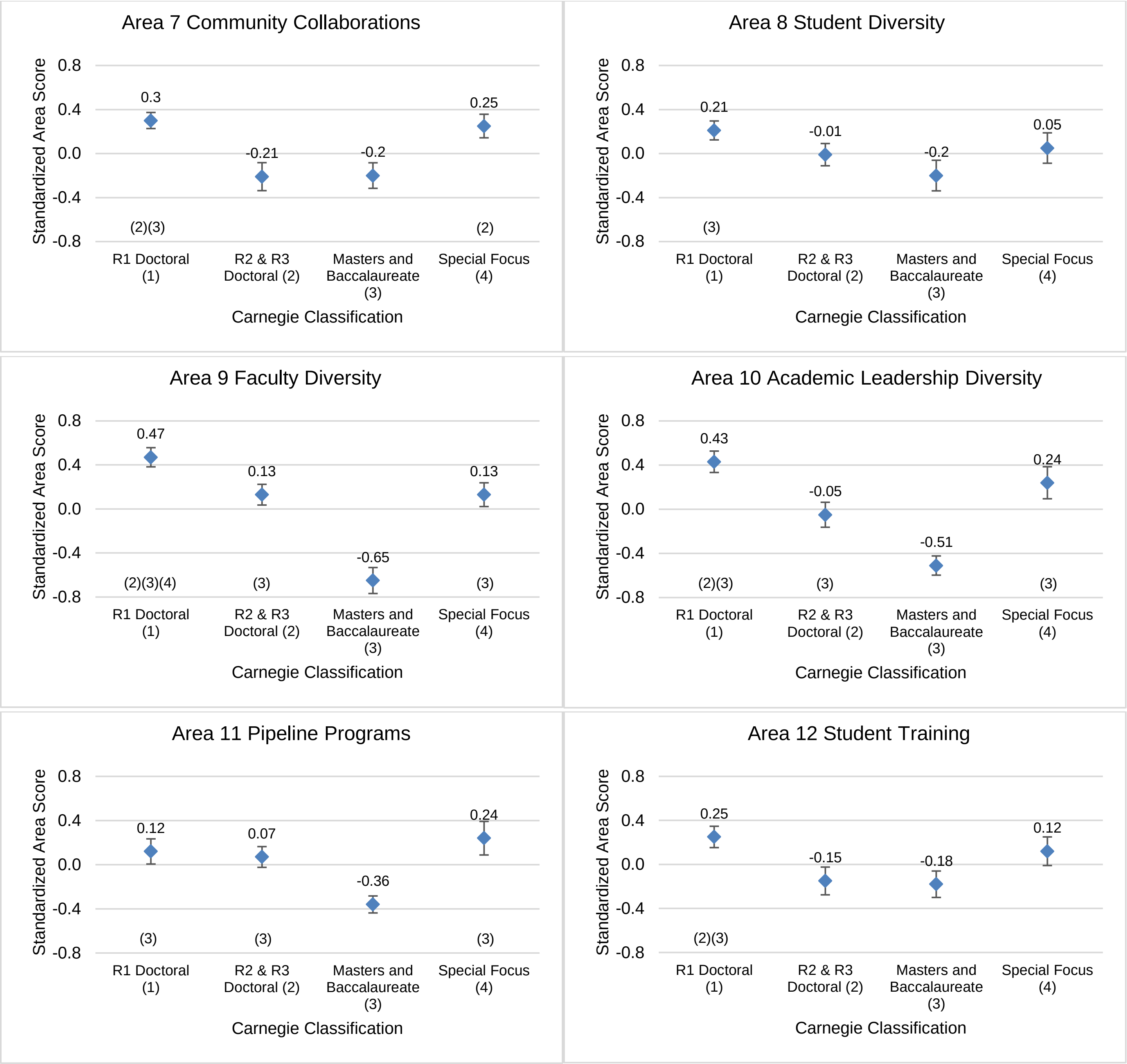

© 2022 Batra S et al. *JAMA Network Open*.

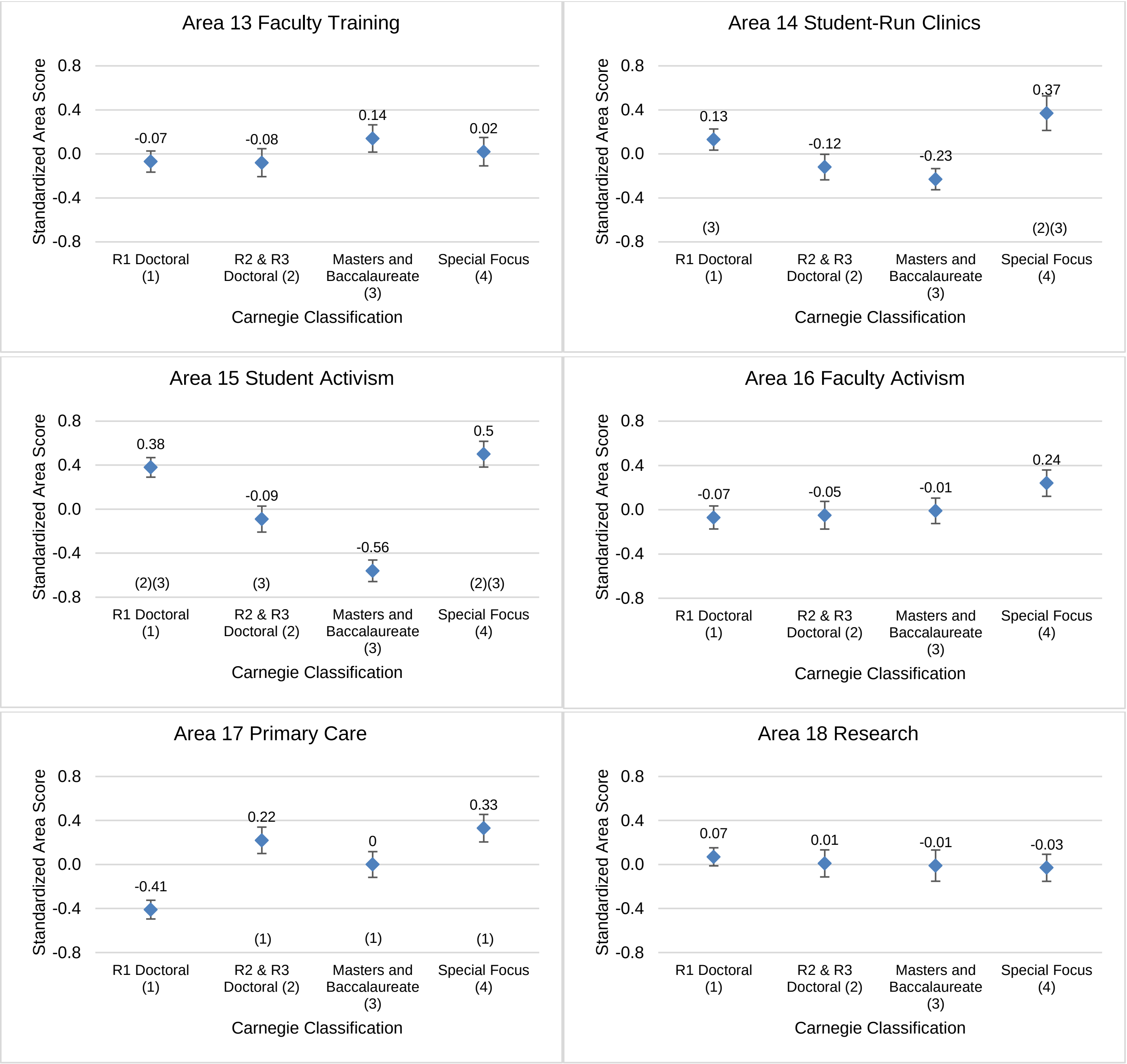

[a](1)(2)(3)(4)(5) number indicate the four Carnegie classification groups. Numbers in parentheses above the x axis indicate the groups whose means are significantly LOWER than this group's mean, based on pairwise t-tests with a finite population correction and Fisher's LSD correction. For example, in Area 15 (Student Activism), groups 1 (R1 Doctoral), and 4 (Special Focus) score significantly higher than groups 2 (R2 &R3 Doctoral), and 3 (Masters and Baccalaureate).

[b]Institutional classification is based on the Carnegie Classification of Institutions of Higher Education, 2018

[c]There were 67 R1 Doctoral, 58 R2 & R3 Doctoral, 68 Masters and Baccalaureate, and 44 Special Focus institutions included in the analysis.

© 2022 Batra S et al. *JAMA Network Open*.
